# Switching the Post-translational Modification of Translation Elongation Factor EF-P

**DOI:** 10.3389/fmicb.2019.01148

**Published:** 2019-05-24

**Authors:** Wolfram Volkwein, Ralph Krafczyk, Pravin Kumar Ankush Jagtap, Marina Parr, Elena Mankina, Jakub Macošek, Zhenghuan Guo, Maximilian Josef Ludwig Johannes Fürst, Miriam Pfab, Dmitrij Frishman, Janosch Hennig, Kirsten Jung, Jürgen Lassak

**Affiliations:** ^1^Center for Integrated Protein Science Munich, Department of Biology I, Microbiology, Ludwig-Maximilians-Universität München, Munich, Germany; ^2^Structural and Computational Biology Unit, European Molecular Biology Laboratory, Heidelberg, Germany; ^3^Department of Bioinformatics, Wissenschaftszentrum Weihenstephan, Technische Universität München, Freising, Germany; ^4^St. Petersburg State Polytechnic University, Saint Petersburg, Russia; ^5^Faculty of Biosciences, Collaboration for Joint PhD Degree Between EMBL and Heidelberg University, Heidelberg, Germany; ^6^Molecular Enzymology Group, University of Groningen, Groningen, Netherlands

**Keywords:** IF5A, EarP, EpmA, bacterial two-hybrid, glycosylation, TDP-rhamnose, *Pseudomonas aeruginosa*, NleB

## Abstract

Tripeptides with two consecutive prolines are the shortest and most frequent sequences causing ribosome stalling. The bacterial translation elongation factor P (EF-P) relieves this arrest, allowing protein biosynthesis to continue. A seven amino acids long loop between beta-strands β3/β4 is crucial for EF-P function and modified at its tip by lysylation of lysine or rhamnosylation of arginine. Phylogenetic analyses unveiled an invariant proline in the -2 position of the modification site in EF-Ps that utilize lysine modifications such as *Escherichia coli*. Bacteria with the arginine modification like *Pseudomonas putida* on the contrary have selected against it. Focusing on the EF-Ps from these two model organisms we demonstrate the importance of the β3/β4 loop composition for functionalization by chemically distinct modifications. Ultimately, we show that only two amino acid changes in *E. coli* EF-P are needed for switching the modification strategy from lysylation to rhamnosylation.

## Introduction

Protein biosynthesis is a universally conserved three-step process that occurs on ribosomes and provides a platform for tRNA mediated amino acid delivery. During translation elongation aminoacyl-tRNAs bind to the ribosomal A-site and peptide bond formation is mediated by a peptidyl-tRNA located in the P-site. Relocation of the P-site tRNA to the E-site enables its exiting from the ribosome. The speed of incorporating amino acids into the growing polypeptide chain varies and strongly depends on their chemical nature (Pavlov et al., 2009). Due to its rigid structure, proline in particular delays the peptidyl transfer reaction, being both a poor A-site donor and P-site acceptor substrate (Muto and Ito, 2008; Wohlgemuth et al., 2008; Pavlov et al., 2009; Johansson et al., 2011; Doerfel et al., 2013, 2015). When translating stretches of two or more prolines, ribosomes become arrested (Tanner et al., 2009; Doerfel et al., 2013; Gutierrez et al., 2013; Hersch et al., 2013; Peil et al., 2013; Ude et al., 2013; Woolstenhulme et al., 2013, 2015; Elgamal et al., 2014; Starosta et al., 2014a). Thus consecutive prolines are disfavored in evolution (Qi et al., 2018). However, the structural benefits of polyproline sequences in proteins (Adzhubei et al., 2013; Starosta et al., 2014b) seem to outweigh the translational drawback and favored the evolution of a specialized universal elongation factor termed e/aIF5A in eukaryotes/archaea and EF-P in bacteria (Doerfel et al., 2013; Gutierrez et al., 2013; Ude et al., 2013). Upon polyproline mediated stalling e/aIF5A and EF-P are recruited to the ribosome to a location between the P- and E-tRNA binding sites (Blaha et al., 2009; Saini et al., 2009; Melnikov et al., 2016a,b; Schmidt et al., 2016; Huter et al., 2017).

**FIGURE 1:**
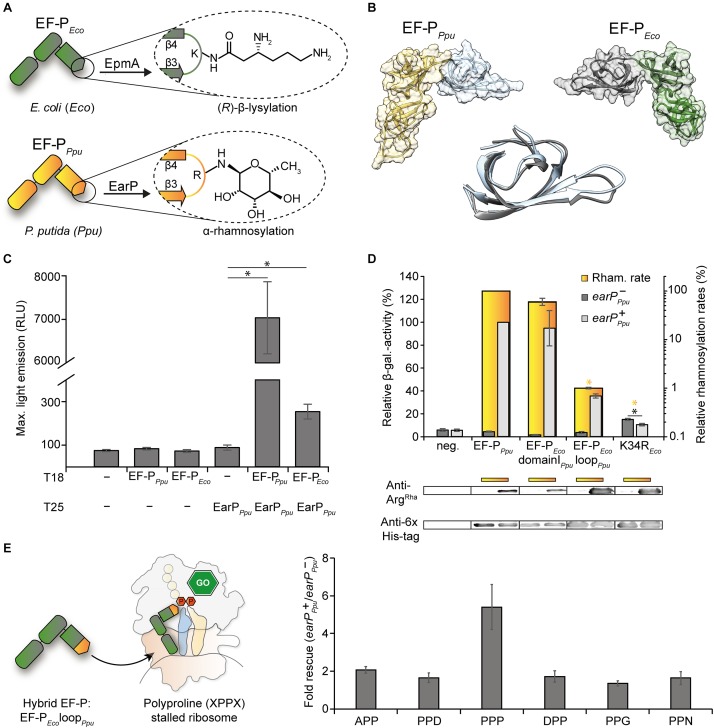
Cross-interaction, -modification and -functionalization studies on *E. coli* EF-P, and EarP of *P. putida* **(A)** Post-translational modifications (PTM) present either in *E. coli* EF-P ((*R*)-β-lysylation) or *P. putida* EF-P (α-rhamnosylation). The modifying enzymes EpmA and EarP are indicated. **(B)** Structural comparison of EF-P from *P. putida* (fold recognition model; see section “Materials and Methods,” left) and *E. coli* (3A5Z:H, right) (Katz et al., 2014). The KOW-like EF-P N-domains are depicted in blue and gray, the OB-folding domains are depicted in orange and green, respectively. Middle: Structural alignment of the KOW-like EF-P N-domains. **(C)** *In vivo* interaction analysis of a T25-EarP*_Ppu_* fusion with T18 fusions of EF-P*_Ppu_* (cognate EarP interaction partner) and EF-P*_Eco_* (non-cognate EarP interaction partner). The maximal light emission from a 40 h time course experiment is given in RLU. 95% confidence intervals of at least six replicates are shown. Asterisks indicate significant (*P* < 0.05) differences in the maximal light emission between cells expressing only one or none of the interaction partners and those co-expressing the corresponding interaction partners. **(D)** Top: *In vitro* rhamnosylation rates (yellow) and *in vivo* ribosome rescue activity (gray) of EF-P*_Eco_*_/_*_Ppu_* variants. In yellow depicted are the relative *in vitro* rhamnosylation rates of EF-P*_Ppu_* (set to 100%) and EF-P*_Eco/Ppu_* variants. Yellow underscoring indicates that these variants were tested *in vitro*. Yellow asterisks indicate significant (*P* < 0.05) differences in the rhamnosylation rate of corresponding variants to the wildtype. *In vivo* measurements were performed in the *E. coli* reporter strain (MG-CR-*efp*-KanS). *E. coli*Δ*efp* cells were complemented either with plasmid encoded His_6_-tagged EF-P_Eco_ variants solely (*earP^-^_Ppu_*, dark gray bars) or in combination with EarP*_Ppu_* (*earP*^+^*_Ppu_*, light gray bars). β-galactosidase activities are given in relative MU with the wild-type EF-P*_Ppu_* in *earP*^+^ cells set to 100%. Rhamnosylation of EF-P_Eco_ was confirmed by Western blot analysis using Anti-rhamnosylarginine specific antibodies (Anti-Arg^Rha^). EF-P*_Eco_* domainI*_Ppu_* was generated by replacing the EF-P KOW-like N-domain I of *E. coli* with the one from *P. putida*. EF-P*_Eco_* loop*_Ppu_* was generated by replacing β3Ωβ4 from *E. coli* with the corresponding β3Ωβ4 from *P. putida*. Black asterisks indicate significant (*P* < 0.05; *P* > 0.001) differences in the ribosome rescue activity in the presence (dark gray) and absence (light gray) of EarP. Bottom: *In vivo* EF-P rhamnosylation levels were visualized using Anti-Arg^Rha^ specific antibodies. Corresponding EF-P protein levels were detected with Anti-6×His^®^ **(E)** Effect of the EF-P*_Eco_* loop*_Ppu_* variant on different polyproline containing stalling motifs. Measurements were performed in *E. coli*Δ*efp* cells (JW4107), harboring plasmid encoded the EF-P*_Eco_* loop*_Ppu_* variant in combination with the *lacZ* reporter preceded by different stalling motifs (pBBR1MCS-3 XPPX *lacZ*) in the presence/absence of EarP*_Ppu_*.

With its three domains (Figure 1A,B), EF-P spans both ribosomal subunits and forms an L-shaped, tRNA mimicking structure (Hanawa-Suetsugu et al., 2004; Katz et al., 2014). Whereas the two OB-Folding domains (Oligonucleotide Binding) II and III are likely to be involved in P-site tRNA^Pro^ (Katoh et al., 2016) and E-site codon (Huter et al., 2017) recognition, the EF-P KOW-like N-domain I is crucial for the catalytic activity. Specifically, a seven amino acid long apical loop region between beta-strands three and four (β3Ωβ4) protrudes toward the peptidyl transferase center (Blaha et al., 2009; Huter et al., 2017). A conserved positively charged residue at the loop tip mediates stabilization and positioning of the CCA-end of the P-site tRNA^Pro^ in a way favorable for peptide bond formation (Doerfel et al., 2013, 2015; Lassak et al., 2015). EF-P activity is further enhanced by post-translational extensions of this specific tip residue (Doerfel et al., 2013; Lassak et al., 2015). Interestingly the underlying bacterial modifications appear to be chemically diverse (Lassak et al., 2016; Figure 1A). In a subset of bacteria including the Gram-negative model organism *Escherichia coli*, a lysine residue K34 is β-lysylated (Bailly and de Crecy-Lagard, 2010; Navarre et al., 2010; Yanagisawa et al., 2010) with (*R*)-β-lysine (Behshad et al., 2006) at the 𝜀-amino group, employing the catalytic activity of the EF-P specific ligase EpmA (YjeA, PoxA, GenX) (Roy et al., 2011). Subsequent hydroxylation by EpmC (YfcM) (Peil et al., 2012; Kobayashi et al., 2014) presumably at the (*R*)-β-lysyl-lysine C5 atom (Huter et al., 2017) completes the modification, but is negligible for function (Bullwinkle et al., 2013). A chemically related amino acid – 5-amino-pentanolyl-lysine – was found on *Bacillus subtilis* EF-P (Rajkovic et al., 2016). By contrast, activity of a distinct EF-P subset encoded in the β-proteobacterial subdivision and certain γ-proteobacteria such as *Pseudomonas putida* and *Shewanella oneidensis* depends on α-rhamnosylation of arginine at the equivalent position (Lassak et al., 2015; Rajkovic et al., 2015; Yanagisawa et al., 2016). This glycosylation is mediated by the GT-B folding glycosyltransferase EarP (Krafczyk et al., 2017; Sengoku et al., 2018) belonging to the enzyme family GT104 according to the CAZy database (Coutinho et al., 2003).

Despite their distinct chemical nature both lysine as well as arginine modifications of EF-P promote proline-proline peptide bond formation at the ribosome. We therefore asked whether there is a specific conservation pattern around the modified residue of diverse EF-Ps and if so how such a specific context contributes to modification efficiency and ribosome rescue. Using bioinformatics and site directed mutagenesis, we were able to show that EarP mediated modification of *E. coli* EF-P requires only the substitution of the protruding lysine by arginine. However, this protein derivative remained translationally inactive. Notably, we recognized a selective pressure on the amino acid located at the second position N-terminal of the modification site. While bacteria encoding EF-P with protruding lysine contain an invariant proline, those with arginine instead strictly select against it. Strikingly, the additional substitution of this residue in this context in *E. coli* EF-P led to a variant that even promotes peptide bond formation in polyproline arrested ribosome upon arginine rhamnosylation. We therefore reason that the presence or absence of this specific proline orients β3Ωβ4 in a way that results in translationally active EF-Ps with modifications similar to either (*R*)-β-lysylation or α-rhamnosylation.

## Materials and Methods

### Plasmid and Strain Construction

All strains, plasmids and oligonucleotides used in this study are listed and described in the Supplementary Tables S1–S3. All kits and enzymes were used according to manufacturer’s instructions. Plasmid DNA was isolated using the Hi Yield^®^ Plasmid Mini Kit from Süd Laborbedarf. DNA fragments were purified from agarose gels using the Hi Yield^®^ Gel/PCR DNA fragment extraction kit from Süd Laborbedarf. All restriction enzymes, DNA modifying enzymes and the Q5^®^ high fidelity DNA polymerase for PCR amplification were purchased from New England BioLabs.

*Escherichia coli* strain KV1 for bacterial two-hybrid analysis was constructed as follows: The *luxCDABE* operon from *Photorhabdus luminescens* was amplified from pBAD/HisA-Lux (Volkwein et al., 2017) and integrated into *E. coli* LF1 as essentially described previously by Fried et al. (2012). To keep the ability of blue/white screening, a synthetic ribosomal binding site predicted by RBS calculator (Salis et al., 2009; Espah Borujeni et al., 2014) was introduced upstream of the *lacZ* start site. Afterward *cyaA* was deleted using Red^®^/ET^®^ recombination technology and the kanamycin cassette was removed using the 709-FLPe/amp expression vector in accordance to the Quick and Easy *E. coli* Gene Deletion Kit (Gene Bridges, Germany). In the same way, *epmA_Eco_* was deleted in the *E. coli* Δ*efp* reporter strain MG-CR-efp-KanS, resulting in the Δ*efp_Eco_/*Δ*epmA_Eco_* reporter strain MG-CR-efp-epmA-KanR. The Δ*efp_Eco_* reporter strain MG-CR-efp-KanS itself was generated by removing the kanamycin resistance cassette from MG-CR-efp (Lassak et al., 2015) using also the Quick and Easy *E. coli* Gene Deletion Kit of Gene Bridges according to the manufacturer’s instructions.

### Growth Conditions

*Escherichia coli* cells were routinely grown in Miller modified Lysogeny Broth (LB) (Bertani, 1951; Miller, 1972; Bertani, 2004) at 37°C aerobically under agitation, if not indicated otherwise. When required, media were solidified by using 1.5% (w/v) agar. The medium was supplemented with antibiotics at the following concentrations when indicated: 100 μg/ml ampicillin sodium salt, 50 μg/ml kanamycin sulfate, 30 μg/ml chloramphenicol, or 15 μg/ml tetracycline hydrochloride. Plasmids carrying the P*_BAD_* promoter (Guzman et al., 1995) were induced with L-arabinose at a final concentration of 0.2% (w/v).

### SDS-PAGE and Western Blotting

For protein analyses cells were subjected to 12% (w/v) sodium dodecyl sulfate (SDS) polyacrylamide gel electrophoresis (PAGE) as described by Laemmli (1970). To visualize and confirm protein separation, 2,2,2-trichloroethanol was incorporated into the polyacrylamide gels (Ladner et al., 2004) and detected within a Gel Doc^TM^ EZ gel documentation system (Bio-Rad). Afterward the proteins were transferred onto nitrocellulose membranes (Whatman) which were then subjected to immunoblotting. In a first step the membranes were incubated either with 0.1 μg/mL Anti-6×His^®^ antibody (Abcam, Inc.) to detect EF-P, or with 0.25 μg/ml Anti-Arg^Rha^ antibody (Li et al., 2016; Krafczyk et al., 2017) to visualize rhamnosylation. These primary antibodies (rabbit) were then targeted with 0.2 μg/ml Anti-rabbit alkaline phosphatase-conjugated secondary antibody (Rockland). Localization was visualized by adding development solution [50 mM sodium carbonate buffer, pH 9.5, 0.01% (w/v) p-nitro blue tetrazolium chloride (NBT) and 0.045% (w/v) 5-bromo-4-chloro-3-indolyl-phosphate (BCIP)].

### β-Galactosidase Activity Assay

*Escherichia coli* Δ*efp* (MG-CR-efp-KanS) or Δ*efp/*Δ*epmA* (MG-CR-efp-epmA-KanR) reporter strain cells, in which *lacZ* expression is controlled by the *cadBA* promoter, were grown overnight (o/n) in 100 mM sodium-phosphate buffered Miller modified LB (pH 5.8) under microaerobic conditions and with agitation at 37°C. On the next day, cells were harvested by centrifugation, and the β-galactosidase activities were determined as described (Tetsch et al., 2008) and are given in relative Miller units (MU) (Miller, 1992).

Whenever the plasmid based reporter system pBBR1MCS-3 XPPX *lacZ* (Peil et al., 2013) was used, cells were grown o/n in 100 mM sodium-phosphate buffered Miller modified LB (pH 5.8), microaerobically under agitation at 37°C. Whenever the *E. coli* Δ*epmA* reporter strain (MG-CL-12-yjeA) (Ude, 2013) was used, cells were grown in potassium buffered KE minimal medium (Epstein and Kim, 1971) pH 5.8, supplemented with 10 mM lysine, 0.2% glycerol and antibiotics in the appropriate concentrations. Whenever *efp_Ppu_* and *earP_Ppu_* were co-expressed from pBBR1MCS2 (Kovach et al., 1995) and pBAD33, respectively, cells were grown in 100 mM sodium-phosphate buffered Miller modified LB (pH 5.8), microaerobically under agitation at 30°C. Whenever *efp_Ppu_* and *earP_Ppu_* were co-overexpressed from pBAD24 and pBAD33, respectively, cells were grown in 100 mM sodium-phosphate buffered Miller modified LB (pH 5.8) and 20 mM arabinose, aerobically under agitation at 30°C. In all cases, the cells were harvested by centrifugation on the next day, and the β-galactosidase activities were determined as described (Tetsch et al., 2008) and are given in MU (Miller, 1992).

### NMR Experiments

To obtain labeled proteins for NMR studies, bacterial overproductions were performed in M9 glucose minimal medium (Miller, 1972) containing either ^15^N-labeled ammonium chloride alone (pET-SUMO-*efp_Eco_*K34R, pET-SUMO-*efp_Eco_* P32S, pET-SUMO-*efp_Eco_* P32S K34R, pET-SUMO-*efp_Ppu_*), or ^15^N-labeled ammonium chloride in combination with ^13^C labeled glucose (pET-SUMO-*efp_Eco_*, pET-SUMO-*efp_Eco_* loop*_Ppu_*). Overproduction of these N-terminally His_6_-SUMO tagged hybrid EF-P variants was induced in *E. coli* BL21 (DE3) by the addition of 1 mM isopropyl β-D-1-thiogalactopyranoside (IPTG; Sigma Aldrich) during exponential growth. Until the induction point the cells were grown at 37°C, after IPTG induction the temperature was shifted to 18°C and the cells were grown o/n. On the next day, the cells were harvested by centrifugation. The resulting pellet was resuspended on ice in dialysis buffer 1 (100 mM Na_2_HPO_4_/NaH_2_PO_4_, pH 6.5, 1 mM DTT). Cells were lysed using a continuous-flow cabinet from Constant Systems Ltd., at 1.35 kbar, in combination with sonication. The resulting lysate was centrifuged for 40 min at 4°C at 39,810 ×*g*. The His_6_-SUMO tagged proteins were purified in a first step with nickel-nitrilotriacetic acid (Ni-NTA; Qiagen) according to the manufacturer’s instructions, using 20 mM imidazole for washing and 250 mM imidazole for elution. Subsequently, imidazole was removed by dialysis o/n at 4°C in dialysis buffer 1. Afterward, the His_6_-SUMO tag was cleaved off using His_6_-Ulp1 protease (Starosta et al., 2014b), followed by a second Ni-NTA purification step to remove the His_6_-SUMO tag itself as well as the His_6_ tagged Ulp1 protease. As a final step, the purified protein was dialyzed again o/n at 4°C in dialysis buffer 1.

C-terminally His_6_-tagged EarP*_Ppu_* for NMR interaction studies was overproduced in *E. coli* LMG194 cells harboring a pBAD33-*earP_Ppu_* plasmid in Miller modified LB at 37°C. During exponential growth, 0.2% (w/v) L-arabinose was added. After induction, the temperature was shifted to 18°C, and the cells were grown o/n. On the next day, the cells were harvested by centrifugation. The resulting pellet was resuspended on ice in dialysis buffer 2 (100 mM Na_2_HPO_4_/NaH_2_PO_4_, pH 7.5, 50 mM NaCl, 5 mM DTT). Cell lysis, centrifugation of the lysate and the first Ni-NTA purification step was performed as described above. In a final step, the purified protein was dialyzed o/n in dialysis buffer 2 to remove imidazole from the purification step.

All ^15^N NMR relaxation experiments for EF-P and its variants were performed in 100 mM Na_2_HPO_4_/NaH_2_PO_4_, pH 6.5 and 1 mM DTT. NMR data were recorded at 298 K for ∼ 0.15–0.18 mM of EF-P*_Eco_* and its variants except for EF-P*_Eco_* P32S for which the data were recorded at 0.09 mM due to low yields of expression. Pulse experiments were performed on an 800 MHz Bruker Avance III NMR spectrometer equipped with a TXI cryogenic probehead. Amide ^15^N relaxation data of *R*_1_, *R*_2_, and steady-state heteronuclear {^1^H}–^15^N-NOE experiments were performed as described before (Farrow et al., 1994; Korzhnev et al., 2002). *T*_1_ data were measured with 11 different relaxation delays: 20, 50, 100, 150, 250, 400, 500, 650, 800, 1000, and 1300 ms, whereby 150 ms was used as duplicate. *T*_2_ data were determined by using eight different relaxation delays: 16, 32, 48, 64, 80. 96, 112, and 128 ms using 16 ms as duplicate. Duplicate time points were used for error estimation. The correlation time (*τ*_c_) of the protein molecule was estimated using the ratio of averaged *T*_2_/*T*_1_ values (Farrow et al., 1994). Steady-state heteronuclear {^1^H}–^15^N-NOE experiments were recorded with and without 3 s of ^1^H saturation. All relaxation experiments were acquired as pseudo-3D experiments. The spectra were processed with NMRPipe (Delaglio et al., 1995) and peak integration and relaxation parameter calculation was performed using PINT (Niklasson et al., 2017).

For the titration of EF-P*_Eco_* and its variants with 2× EarP*_Ppu_*, both the proteins were dialyzed against 100 mM Na_2_HPO_4_/NaH_2_PO_4_, pH 7.5, 50 mM NaCl, and 1 mM DTT. Experiments were recorded on an 800 MHz Bruker NMR spectrometer equipped with a TXI cryogenic probehead at 298 K. Protein backbone assignments for EF-P*_Eco_* and EF-P*_Eco_* loop*_Ppu_* were obtained from HNCACB, CBCA(CO)NH, and HNCA experiments (Sattler et al., 1999). Data analysis was performed in CcpNmr Analysis software (Vranken et al., 2005). Resonance assignments of EF-P variants have been deposited at the BMRB with the following accession codes: 27811.

### *In vitro* Rhamnosylation Studies

To obtain EF-P variants for *in vitro* rhamnosylation studies, protein overproductions were performed in *E. coli* LMG194 cells, grown in Miller modified LB, harboring the following C-terminally His_6_-tagged EF-P constructs (See also Supplementary Table S2):

• EF-P*_Ppu_*: pBAD24-*efp_Ppu_*, pBAD24-*efp_Ppu_* S30P, pBAD24-*efp_Ppu_* G31A, pBAD24-*efp_Ppu_* R32K, pBAD24-*efp_Ppu_* N33G, pBAD24-*efp_Ppu_* S34Q
• EF-P*_Eco_*: pBAD24-*efp_Eco_* K34R, pBAD24-*efp_Eco_* P32S K34R, pBAD24-*efp_Eco_* K34R G35N, pBAD24-*efp_Eco_* K34R Q36S, pBAD24-*efp_Eco_* P32S K34R G35N, pBAD24-*efp_Eco_* P32S K34R Q36S, pBAD24-*efp_Eco_* K34R G35N Q36S, pBAD24-EF-P_Eco_ loop_Ppu_, pBAD24-EF-P_Eco_ domainI_Ppu_

Furthermore, C-terminally His_6_ tagged EarP*_Ppu_* was overproduced in *E. coli* LMG194 harboring pBAD33-*earP_Ppu_*.

To overproduce proteins, cells harboring corresponding plasmids were grown at 37°C, and during exponential growth, 0.2% (w/v) L-arabinose was added to induce protein production. After induction, the temperature was shifted to 18°C, and the cells were grown o/n. On the next day, the cells were harvested by centrifugation and the resulting pellet was resuspended in buffer 3 (100 mM Na_2_HPO_4_/NaH_2_PO_4_, pH 7.5, 50 mM NaCl). Cells were then lysed by sonication and the resulting cell lysate was clarified by centrifugation for 40 min at 4°C at 39,810 ×*g*. The His_6_ tagged proteins were then purified using Ni-NTA beads (Qiagen) according to the manufacturer’s instructions, whereby 20 mM imidazole was used for washing and 250 mM imidazole for elution of the His_6_ tagged proteins. Subsequently, imidazole was removed by dialysis o/n at 4°C in buffer 3, followed by a second dialysis step at the next morning for 5 h, again in buffer 3. The resulting proteins were then used for *in vitro* rhamnosylation assays.

Kinetic parameters were determined by varying TDP-β-L-rhamnose (TDP-Rha) concentrations while keeping concentrations of EarP*_Ppu_* (0.1 μM) and unmodified EF-P*_Ppu_* (2.5 μM) constant. A mixture of EarP*_Ppu_* and unmodified EF-P*_Ppu_* was equilibrated to 30°C in 100 mM Na_2_HPO_4_/NaH_2_PO_4_, pH 7.6. The reaction was started by the addition of TDP-Rha and was stopped after 20 s of incubation at 30°C by the addition of one volume twofold Laemmli buffer (Laemmli, 1970) and incubation at 95°C for 5 min. Samples were subjected to SDS-PAGE and rhamnosylated EF-P*_Ppu_* was detected using an Anti-Arg^Rha^ antibody (Krafczyk et al., 2017). A secondary FITC coupled Anti-rabbit antibody (Abcam, United Kingdom) was used to visualize rhamnosylation in a LI-COR Odyssey CLx. Band intensities were quantified using ImageJ (Schneider et al., 2012). *K_m_* values were determined by fitting relative band intensities to the Michaelis–Menten equation using SigmaPlot. The *K_m_* of 5 μM TDP-Rha was determined using commercially available substrate (Carbosynth, United Kingdom). Previously, we determined a *K_m_* of 50 μM using biochemically synthesized TDP-Rha (Krafczyk et al., 2017). After rigorous assessment of this discrepancy we found that contaminations with ammonium acetate were responsible for a miscalculation of the TDP-Rha concentration in stock solutions.

*In vitro* rhamnosylation of EF-P*_Eco_* and EF-P*_Ppu_* variants was conducted in 100 mM Na_2_HPO_4_/NaH_2_PO_4_, pH 7.5 containing 50 mM NaCl. A master mix containing 25 μM of the corresponding EF-P variant and 100 μM TDP-Rha was prepared in a reaction tube and divided into 10 μl aliquots. The reaction was started by addition of 10 μl of 0.5 μM EarP solution and stopped after distinct time intervals by addition of 20 μl twofold Laemmli buffer and immediate heating to 95°C in an Eppendorf ThermoMixer for 2 min. All samples were diluted by a factor of 10 in onefold Laemmli buffer and 20 μl (corresponding to 0.5 μg of EF-P) were subjected to SDS-PAGE and Western blotting. Rhamnosylated EF-P was detected and visualized using a polyclonal rabbit Anti-Arg^Rha^ and a fluorescence labeled Anti-rabbit antibody, respectively. Band intensities were determined using ImageJ (Schneider et al., 2012). Relative rhamnosylation rates were calculated by plotting the normalized linear range (intensity t_x_/intensity_max_) of the time course and determining the slope of the resulting graphs.

### Isoelectric Focusing

To investigate lysylation of *E. coli* EF-P C-terminally His_6_-tagged EF-P*_Eco_* was overproduced in *E. coli* BW25113 and *E. coli* BW25113 Δ*epmA* cells, harboring the pBAD33-*efp*-His_6_ plasmid, and were grown in Miller modified LB at 37°C. Furthermore, *E. coli* BW25113 was transformed with pBAD33-*efp*-His_6_-*epmAB* to produce post-translationally modified EF-P. During exponential growth, 0.2% (w/v) L-arabinose was added to induce protein production and cells were grown o/n at 18°C. On the next day, cells were harvested by centrifugation. The resulting pellet was resuspended on ice in HEPES buffer (50 mM HEPES, 100 mM NaCl, 50 mM KCl, 10 mM MgCl_2_, 5% (w/v) glycerol, pH 7.0). Cells were lysed using a continuous-flow cabinet from Constant Systems Ltd., at 1.35 kb. The resulting lysates were clarified by centrifugation for 1.5 h at 4°C at 234,998 ×*g*. The His_6_-tagged proteins were purified with Ni-NTA according to the manufacturer’s instructions, using 20 mM imidazole for washing and 400 mM imidazole for elution. In a final step, the purified protein was dialyzed o/n in HEPES buffer to remove imidazole from the purification step.

For isoelectric focusing 0.5 μg of protein per lane was loaded on a native vertical isoelectric focusing gel with a pH gradient range of 4–7 (SERVAGel^TM^) containing approximately 3% (v/v) SERVALYT^TM^. Prior to loading, samples were mixed with twofold IEF sample buffer according to the manufacturer’s instructions and wells were rinsed with SERVA IEF Cathode buffer. Focusing was conducted for 1 h at 50 V, 1 h at 300 V and finally bands were sharpened for 30 min at 500 V. Western blotting was conducted as described above using 0.1 μg/ml Anti-EF-P*_Eco_* (Eurogentec).

### Bacterial Two-Hybrid Analysis

Protein-protein interactions were detected using the bacterial adenylate cyclase two-hybrid system kit (Euromedex) according to the manufacturer’s instructions. This system is based on functional reconstitution of split *Bordetella pertussis* adenylate cyclase CyaA, which in turn activates the *lac* promoter P*_lac_* being dependent on the cAMP receptor protein CAP (Supplementary Figure S1A). The *E. coli* KV1 strain used in this study was generated by start to stop deletion of the *cyaA* gene from *E. coli* LF1 (Fried et al., 2012) and subsequent incorporation of the *lux* operon at the *lac* locus by using described methods. Applicability of this strain was tested by assessing the self-interaction of the GCN4 leucine zipper in *E. coli* KV1 and the commercially available bacterial two-hybrid strains *E. coli* BTH101 (Euromedex) and *E. coli* DHM1 (Euromedex) on X-Gal containing screening plates. For this purpose, the reporter strains were co-transformed with the plasmids pKT25-zip (Euromedex) and pUT18C-zip (Euromedex) that encode for protein hybrids of the leucine zipper and the corresponding CyaA fragment. While all reporter strains respond with comparable β-galactosidase mediated color formation, KV1 exhibits an additional light output (Supplementary Figure S1B). Liquid cultures of transformants containing 50 μg/ml kanamycin sulfate, 100 μg/ml carbenicillin and 0.5 mM IPTG were inoculated from single colonies and incubated at 30°C for 8 h. 2 μl of liquid culture were spotted on LB plates containing 40 μg/ml 5-bromo-4-chloro-3-indolyl-β-D-galactopyranoside (X-Gal) and 0.5 mM IPTG as well as 50 μg/ml kanamycin sulfate and 100 μg/ml carbenicillin. Pictures were taken after 32 h of incubation at 30°C.

For measuring interaction strength between EarP*_Ppu_* and EF-P*_Ppu_* or EF-P*_Eco_*, chemically competent *E. coli* KV1 cells were co-transformed with pKT25-EarP*_Ppu_* and either pUT18C-EF-P*_Ppu_* or pUT18C-EF-P*_Eco_*. Transformants carrying leucine-zipper-reporter hybrids (pUT18-zip and pKT25-zip) were used as positive controls, whereas transformants containing pUT18C and pKT25 vector backbones served as negative controls. Single colonies were picked and used to inoculate 96-well plates, with each well containing 200 μl of LB medium supplemented with 0.5 mM IPTG as well as 50 μg/ml kanamycin sulfate and 100 μg/ml carbenicillin. Plates were incubated at 30°C and under moderate agitation (550 rpm in Eppendorf ThermoMixer) for 16 h. On the next morning, Costar 96 Well White plates containing 200 μl of LB medium (0.5 mM IPTG, 50 μg/ml kanamycin sulfate, 100 μg/ml carbenicillin) were inoculated with 2 μl of o/n culture and luminescence output was monitored in 10-min intervals for 40 h in a Tecan Spark with 240 rpm at 30°C.

### Bioinformatic Analyses

#### Fold Recognition and Comparison of EF-P Structures

The EF-P*_Ppu_* fold recognition model was generated using the online user interface of the I-TASSER server (Zhang, 2008; Roy et al., 2010; Yang et al., 2015). Chain B of the *Pseudomonas aeruginosa* EF-P crystal structure (3OYY:B) (Choi and Choe, 2011) was used as “template without alignment.” The resulting model exhibits a C-score (confidence score for estimating the quality of fold recognition models predicted by I-TASSER. Range: (-5; 2); high values signify a model with a high confidence and vice-versa) of 1.27 and an estimated TM-score [measure for the similarity between the predicted model and the native structures. Range: (0,1), with 1 indicating a perfect match] of 0.89 ± 0.07, indicating high confidence and correct topology. The structural alignment of the N-terminal KOW-like EF-P N-domains of the EF-P*_Ppu_* fold recognition model and *E. coli* EF-P (3A5Z:H) (Katz et al., 2014) was generated using the UCSF Chimera (Pettersen et al., 2004) MatchMaker function (Chain pairing: Best-aligning pair of chains; Alignment algorithm: Needleman–Wunsch; Matrix: Blosum-62; Gap extension penalty: 1; Matching to 2.0 angstroms) and resulted in an RMSD of 1.005 angstroms between 51 pruned atom pairs. Only amino acids 1–63 of each EF-P structure were used for the alignment.

### Sequence Data and Domain Analysis

Using the search query “Bacteria”[Organism] AND [“reference genome”(refseq category) OR “representative genome ”(refseq category)] AND “complete genome”[filter] we obtained from the RefSeq database (O’Leary et al., 2016) a collection of 1644 proteomes corresponding to complete and/or representative genomes. HMMER searches (Finn et al., 2015) against the locally installed Pfam database (Finn et al., 2016) was used to identify domains in proteins using the *hmmscan* *e*-value cut-off of 0.001.

We created an initial dataset of EF-P proteins by considering only those gene products that exclusively contain the three domains of interest – “EFP_N” (KOW-like domain), “EFP” (OB domain), “Elong-fact-P_C” (C-terminal). Subsequently we excluded from the dataset the proteins annotated in the UniProt database (UniProt Consortium, 2018) as YeiP, which are EF-P’s paralogs and possess the same domain architecture. Additional sequence comparisons did not yield any misannotated YeiP proteins.

Sequences of predicted KOW-like domains, in which the β3Ωβ4 loop is located, were aligned using the “E-INS-i” algorithm from the MAFFT software suite (Katoh and Standley, 2013). According to the trimAl tool (Capella-Gutierrez et al., 2009) the MSA of KOW-like domain sequences does not contain any sequences particularly prone to introduce poorly aligned regions. Eleven sequences were manually deleted from the set as they introduce gaps in the multiple alignment of the β3Ωβ4 region (alignment positions 31–37) and the alignment was re-computed. MSA file is available in the Supplementary Materials.

The final EF-P set contains 1166 sequences from 1137 genomes, including 29 genomes containing two EF-P paralogs. Sequence logos were built using ggseqlogo R package (Wagih, 2017).

The EF-P-containing genomes were scanned for the EpmA, EpmC, EarP, DHS, and YmfI proteins. We identified 358, 143, 100, and 128 EpmA, EpmC, EarP, and DHS proteins based, which are single-domain proteins containing the “tRNA-synt_2,” “EpmC,” “EarP” (Lassak et al., 2015) and “DS” (Brochier et al., 2004) domains, respectively. Orthologs of the YmfI protein (*Uniprot ID: O31767*) from *Bacillus subtilis* (Hummels et al., 2017; Rajkovic and Ibba, 2017) were obtained from the orthologous group 508579 of the OMA database (Altenhoff et al., 2018).

### Phylogenetic Analysis

IQ-Tree 1.6.10 (Nguyen et al., 2015) was used to infer a phylogenetic tree of KOW-like domains by the maximum likelihood method, with the LG substitution matrix and the number of standard non-parametric bootstrap replicates set to 100. The tree file in PDF format and its visualization including bootstrap support values are available as Supplementary Dataset S2. Using the *ete3* python package (Huerta-Cepas et al., 2016) the tree was rooted to the midpoint outgroup and converted to ultrametric. The evolutionary reconstruction of ancestral states was performed using the *ace* function from the *phytools* R package (Revell, 2012), which implements the maximum likelihood estimation. We used the *ggtree* R package (Yu et al., 2017) to visualize the evolutionary reconstruction of ancestral states on the tree of KOW-like domains and annotate it with the amino acid located at the 34th position, the presence or absence of a certain modification enzyme, and the taxonomy for Proteobacteria, Firmicutes and Actinobacteria. A tree with a more detailed taxonomic annotation is available in Supplementary Materials.

## Results and Discussion

### K34R Substitution of *E. coli* EF-P Is Sufficient for Non-cognate Rhamnosylation by EarP

Canonically, N-linked protein glycosylation occurs at a consensus sequence motif (Helenius and Aebi, 2004). By contrast, the glycosyltransferase EarP seems to recognize rather the overall shape of domain I of its target EF-P (Krafczyk et al., 2017; Sengoku et al., 2018; Figure 1B). Notably, and despite large sequence diversity all EF-Ps are structurally similar (Yanagisawa et al., 2010; Choi and Choe, 2011; Lassak et al., 2016). In this regard the EF-Ps of the two model organisms *E. coli* (EF-P*_Eco_*) and *P. putida* (EF-P*_Ppu_*) are highly superimposable (Figure 1B) although they share a sequence identity below 30%. We were therefore curious whether cross-interaction between the non-cognate partners EF-P*_Eco_* and EarP*_Ppu_* is possible. To this end we constructed a highly sensitive bacterial two-hybrid *E. coli* reporter strain KV1 to combine it with the plasmid system which was reported previously (Karimova et al., 1998) (Supplementary Figure S1A). Distinct from the original strains we used bioluminescence as a readout (see section “Materials and Methods”) and generated C-terminal fusions of the two complementary fragments T25 and T18 of the *Bordetella pertussis* adenylate cyclase with EarP*_Ppu_* and the EF-Ps of *E. coli* and *P. putida* (T25-EarP*_Ppu_*, T18-EF-P*_Ppu_*, T18-EF-P*_Eco_*). Interaction strength of protein pairs was assessed by determining the maximal light output in a 40 h time course experiment (Supplementary Figure S1B). Cells co-expressing the cognate interaction partners EarP*_Ppu_* and EF-P*_Ppu_* emitted a maximum of 7,000 RLU (Figure 1C). When EarP*_Ppu_* and the non-cognate EF-P*_Eco_* were co-produced, a maximal light emission of 255 RLU was observed. Although this is substantially lower, the measured value is significantly above background levels (maximal RLU of <100) and thus clearly demonstrates cross-interaction of EarP*_Ppu_* and EF-P*_Eco_*.

Knowing that EF-P*_Eco_* and EarP*_Ppu_* do cross-interact, we next assessed whether cross-rhamnosylation also occurs. Therefore we took advantage of a previously introduced rhamnosylarginine specific antibody (Li et al., 2016; Krafczyk et al., 2017) and used it to test *in vitro* rhamnosylation over time. The rhamnosylation efficiency of EarP*_Ppu_* with its cognate partner EF-P*_Ppu_* was determined with 50 μM TDP-β-L-rhamnose (TDP-Rha) (=10-fold *K_m_*; Supplementary Figure S2A) and set to 100%. Next we tested an EF-P*_Eco_* variant in which solely the modification site K34 was changed to arginine (K34R*_Eco_*). However, we did not observe any rhamnosylation *in vitro* (Figure 1D and Supplementary Figure S2B) presumably as a result of suboptimal contacts between EF-P*_Eco_* and EarP*_Ppu_* (Sengoku et al., 2018; Figure 1C). As important interaction sites between EF-P and its corresponding modification system are predominantly located within the first 65 amino acids (Navarre et al., 2010; Yanagisawa et al., 2010; Krafczyk et al., 2017; Sengoku et al., 2018) we now swapped the N-domain of EF-P*_Eco_* with the one from EF-P*_Ppu_* (EF-P*_Eco_* domainI*_Ppu_*). In line with our expectations EF-P*_Eco_* domainI*_Ppu_* was readily modified (Figure 1D). For this reason, in a subsequent step we tested an EF-P*_Eco_* variant with swapped β3Ωβ4 – EF-P*_Eco_* loop*_Ppu_*, containing in total four amino acid substitutions P32S, K34R, G35N, and Q36S. Although the efficiency was strongly reduced (1% compared to EF-P*_Ppu_*) this variant could still be rhamnosylated (Figure 1D).

We previously observed that impairments of EarP variants with largely reduced *in vitro* rhamnosylation rates could be compensated *in vivo* (EF-P modification and functionality) when EF-P and the variants were co-overproduced (Krafczyk et al., 2017; Supplementary Figure S3). This could be explained by an increase of the local protein concentrations within the cells. Consequently, we reinvestigated rhamnosylation of K34R*_Eco_*, EF-P*_Eco_* loop*_Ppu_*, and EF-P*_Eco_* domainI*_Ppu_* by EarP*_Ppu_* in *E. coli*. Following this approach, even the single substituted EF-P*_Eco_* variant reached modification levels comparable to wildtype EF-P*_Ppu_* (Figure 1D, bottom). Thus, we were ultimately able to test these EF-P*_Eco_* variants on their ability to rescue ribosome stalling in an EarP dependent manner. EF-P functionality was measured using a previously established β-galactosidase dependent reporter system (Ude et al., 2013; Figure 1D, top). The assay is based on the effective translation of the polyproline motif containing acid stress responsive transcriptional regulator CadC (Buchner et al., 2015; Schlundt et al., 2017) and activation of its cognate promoter P*_cadBA_* fused to *lacZ* (Ude et al., 2013): β-galactosidase activity is low in *E. coli* cells lacking *efp* but becomes elevated when complementing with both a copy of *earP_Ppu_* and *efp_Ppu_* provided in *trans* (Figure 1D). Similarly, EF-P*_Eco_* domainI*_Ppu_* rescues ribosome stalling upon rhamnosylation, indicating that binding of the two distinct EF-Ps from *E. coli* and *P. putida* to the ribosome occurs presumably at the same position. Hence the structural determinants for proper orientation of the respective protruding residue (lysine or arginine) and accordingly the corresponding modification may predominantly lay in the β3Ωβ4 composition. In line with this assumption rhamnosylated EF-P*_Eco_* loop*_Ppu_* also alleviates the translational arrest occurring at the CadC nascent chain. We note that this rescue activity is not restricted to three consecutive prolines, but encompasses also other diprolyl arrest motifs as demonstrated for APP, DPP, PPD, PPG, and PPN (Figure 1E). In contrast to EF-P*_Eco_* loop*_Ppu_* we measured an unexpected increase in relative β-galactosidase level to about 20% of wildtype activity after introducing K34R*_Eco_* into Δ*efp_Eco_* cells pointing toward a partial complementation of the mutant phenotype. However, this activity was lowered in the concomitant presence of *earP_Ppu_*, which suggests an inhibitory effect of the modification. Presumably, the otherwise preserved EF-P*_Eco_* loop composition in K34R*_Eco_* precludes proper alignment of rhamnosylarginine with the CCA-end of the P-site tRNA^Pro^.

Collectively, these data demonstrate that EarP-mediated rhamnosylation can tolerate substantial changes in the primary sequence of the target protein EF-P. The capability of EF-P to alleviate polyproline dependent translational stalling is, however, strongly affected by changes in β3Ωβ4 sequence. This in turn suggests that both of the modifications rely on a certain acceptor loop architecture that orients the protruding residues in a way favorable for promoting ribosome rescue.

### The Sequence Composition of the EF-P β3Ωβ4 Determines Ribosome Rescue With Distinct Modifications

**FIGURE 2:**
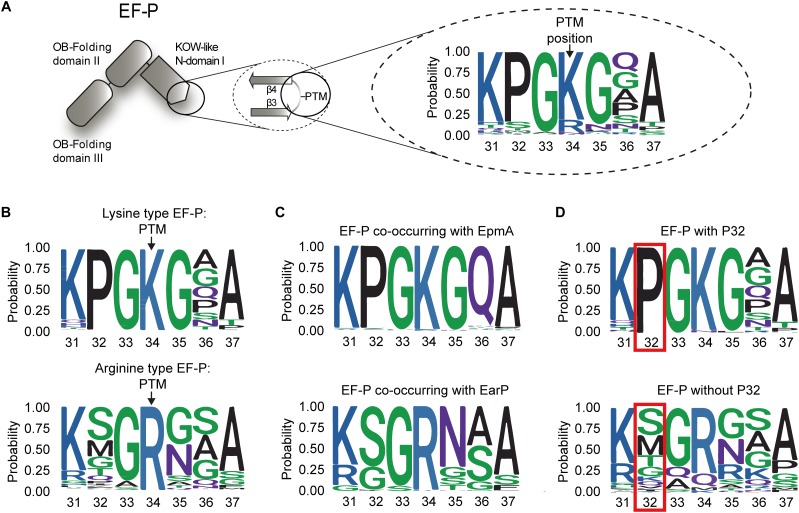
Composition of β3Ωβ4 of the EFP_N superfamily **(A)** Domain architecture of EF-P: The first magnification illustrates β3Ωβ4 of the KOW-like EF-P_N-domain I including the post-translational modification (PTM) site. The second magnification depicts the general amino acid composition of the seven amino acid long loop between the strands β3 and β4 based on 1166 sequences. **(B)** Weblogo of β3Ωβ4 for the EF-Ps with lysine (lysine-type EF-P, upper logo) or an arginine (arginine-type EF-P, lower logo) at position 34 according to the numbering of the *E. coli* ortholog. **(C)** Weblogo of β3Ωβ4 for the EF-Ps of bacteria co-occurring with EpmA (upper logo) or EarP (lower logo). **(D)** Weblogo of β3Ωβ4 for the EF-Ps containing (upper logo) or not containing (lower logo) a proline at position 32 according to the numbering of the *E. coli* ortholog.

Having shown that the EF-P*_Eco_* β3Ωβ4 composition is crucial for rhamnosylation dependent rescue of polyproline arrested ribosomes, we next examined the role of the specific loop amino acids on protein function. Therefore, we initially constructed a phylogenetic tree based on 1166 EF-P sequences. To define modification-specific protein subsets, EpmA and EarP orthologs were collected as described previously (Lassak et al., 2015; Supplementary Dataset S1). The EF-P modification system present in *B. subtilis* was excluded in this study, as the full pathway is still poorly understood (Rajkovic et al., 2016; Witzky et al., 2018). A first sequence logo of β3Ωβ4 numbered according to the *E. coli* protein (amino acids 31 to 37) was generated based on the complete EF-P dataset (Figure 2A and Supplementary Dataset S1). In line with earlier reports (Bailly and de Crecy-Lagard, 2010), the vast majority (81.11%) of EF-Ps have a lysine at the β3Ωβ4 tip (K34), whereas arginine is the second most frequent amino acid occurring in 14.75% of the proteins (Supplementary Dataset S1). The remaining 4.11% contain A (0.51%), M (0.77%), N (2.23%), and Q (0.6%) in this position. We next extracted two subsets of proteins with either a protruding lysine (lysine-type) or arginine (arginine-type) (Figure 2B). This analysis revealed a highly conserved proline in the second position N-terminal of the modification site (P32) in the lysine type subset being almost absent in the arginine-type EF-Ps. Consistently, bacteria with EpmA pathway have this proline in the EF-P sequence whereas those with EarP do not (Figure 2C). With few exceptions the two modification systems thus appear to be mutually exclusive (Lassak et al., 2015; Supplementary Dataset S1). Based on these observations, EF-P sequences were grouped according to the presence or absence of P32 (Figure 2D). Besides lysine (98.73%), we also found that alanine (100%) and asparagine in the protruding position strongly co-occur with proline (100%), whereas other types of amino acids co-occur with P32 extremely rarely or not at all: arginine (2.33%), methionine (0%), and glutamine (0%) (Supplementary Dataset S1).

**FIGURE 3:**
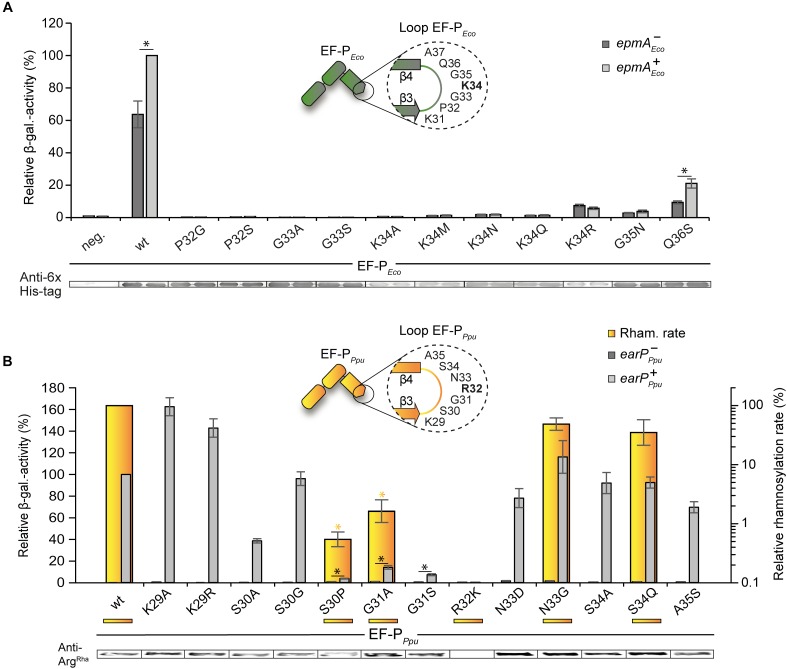
Activity of *E. coli* and *P. putida* EF-P variants with single amino acid substitutions in β3Ωβ4 **(A)** Top: *In vivo* activity measurements of *E. coli* EF-P β3Ωβ4 variants using the described *E. coli* reporter strain. In this strain, either *efp* (*E. coli* MG-CR-efp-KanS, *epmA*^+^ *_Eco_*, dark gray bars) or both, *efp* as well as *epmA* were deleted (*E. coli* MG-CR-efp-epmA-KanR, *epmA*^-^*_Eco_*, light gray bars) and complemented with a plasmid encoded *E. coli* His_6_-tagged EF-P variant. β-galactosidase activities are given in relative MU with the wild-type EF-P*_Eco_* in the *epmA*^+^ background set to 100%. Asterisks indicate significant (*P* < 0.05) differences in the ribosome rescue activity of a given EF-P*_Eco_* variant in the presence of EpmA compared to the same variant in the absence of EpmA. Bottom: Production of EF-P*_Eco_* variants was confirmed by Western blot analysis using Anti-6×His^®^ **(B)** Top: *In vitro* rhamnosylation rates (yellow) and ribosome rescue activity (gray) of EF-P*_Eco_*_/_*_Ppu_* variants. Shown in yellow are the relative *in vitro* rhamnosylation rates of EF-P*_Ppu_* wild type (set to 100%) and EF-P*_Ppu_* amino acid substitution variants. Yellow underscoring depicts that these variants were tested *in vitro*. Yellow asterisks indicate significant (*P* < 0.05) differences in the rhamnosylation rate of corresponding variants to the wildtype. *In vivo* activity measurements of *P. putida* EF-P β3Ωβ4 using the *E. coli* reporter strain (*E. coli* MG-CR-efp-KanS). *E. coli* Δ*efp* cells were complemented either with a plasmid-encoded *P. putida* His_6_-tagged EF-P variant solely (*earP*^-^*_Ppu_*, dark gray bars) or in combination with EarP*_Ppu_* (*earP*^+^*_Ppu_*, light gray bars). β-galactosidase activities are given in relative MU with the wild-type EF-P*_Ppu_* in earP^+^ cells set to 100%. Black asterisks indicate significant (*P* < 0.05; *P* > 0.0001) differences in the ribosome rescue activity of a given EF-P*_Ppu_* variant in the presence of EarP compared to the same variant in the absence of EarP. Bottom: Rhamnosylation levels of EF-P*_Ppu_* variants were detected by Western blot analysis using Anti-Arg^Rha^.

Subsequent to and based on our bioinformatic analysis we mutated β3Ωβ4 of the EF-Ps of both *E. coli* and *P. putida*. EF-P functionality was measured *in vivo*, again using the CadC dependent β-galactosidase reporter system (Ude et al., 2013; Figure 3). The partial P*_cadBA_* activation with plasmid-based K34R*_Eco_* (Figure 1D) intrigued us to first investigate the effect of overproduced unmodified wildtype EF-P*_Eco_*. Therefore, we ectopically expressed *efp_Eco_* in a reporter strain lacking the *E. coli* lysyl ligase EpmA and measured the β-galactosidase outcome (Supplementary Figure S4). Intriguingly, P*_cadBA_* was 50% reactivated compared to a *trans* complementation with *epmA_Eco_*. Presumably, the lysine K34 side chain forms important stabilizing contacts with the CCA-end of the P-site tRNA^Pro^ (Huter et al., 2017), which can in part compensate for a lack of modification. We were therefore next curious whether unmodified amino acids other than lysine and arginine can promote EF-P*_Eco_* functionality without modification. Hence, we substituted K34 by any other amino acid (A, M, N, Q) to be found in the protruding position of β3Ωβ4 of the various EF-Ps (Figure 3A and Supplementary Dataset S1). However, none of the resultant protein variants was translationally active, indicating on the one hand that side chain similarities only between arginine and lysine seems to be high enough to preserve certain of the above-mentioned interactions. On the other hand, the significantly lower activity with K34R*_Eco_* (20%) compared to unmodified EF-P*_Eco_* (50%) points toward a non-stimulating or even negative effect, possibly caused by the guanidino group.

Having demonstrated that substitution of K34 in *E. coli* EF-P is hardly tolerated, we went on to analyze the impact of its context residues. Coherent with its high degree of conservation in lysine-type EF-Ps (Figure 2B,D) an exchange of P32 (P32S*_Eco_*, P32G*_Ec_*_o_) analogous to the arginine-type EF-P sequence logo (Figure 2C) abolishes β-galactosidase activity (Figure 3A). Similarly, a substitution of G33 (G33A*_Eco_*, G33S*_Eco_*) is not tolerated and leads to a loss of function of EF-P*_Eco_*. In comparison, when mutating G35 (G35N*_Eco_*) and Q36 (Q36S*_Eco_*) a residual rescue activity of 3.8 and 21.1%, respectively, is retained. Altogether our analysis of EF-P*_Eco_* β3Ωβ4 unveils important determinants for protein function and thus explains their high degree of conservation.

In our complementary analysis with the EF-P of *P. putida* KT2440 we generated the substitution variants K29R*_Ppu_*, S30P*_Ppu_*, R32K*_Ppu_*, N33G*_Ppu_*, and S34Q*_Ppu_* according to amino acids predominantly found in the lysine-type sequence logo (Figure 2B). We also constructed K29A*_Ppu_*, S30A*_Ppu_*, S30G*_Ppu_*, G31A*_Ppu_*, G31S*_Ppu_*, N33D*_Ppu_*, S34A*_Ppu_*, and A35S*_Ppu_* to further study the impact of the corresponding positions on EF-P activity and rhamnosylation efficiency. An *in vitro* time course analysis was performed (Figure 3B) with wild-type EF-P*_Ppu_* as well as its variants S30P*_Ppu_*, G31A*_Ppu_*, R32K*_Ppu_*, N33G*_Ppu_*, and S34Q*_Ppu_*. This revealed relative rhamnosylation rates with S30P*_Ppu_* and G31A*_Ppu_* (<1% of wild-type activity) being slowest, while N33G*_Ppu_* and S34Q*_Ppu_* reach 62 and 12% compared to wild-type EF-P*_Ppu_*, respectively (Figure 3B). Corresponding to the *E. coli* EF-P variants we also assessed the capability of EF-P*_Ppu_* in alleviating the translational arrest on consecutive prolines *in vivo*. As for the cross-modified K34R*_Eco_* (Figure 1D), we found that overproduction of EarP compensates for reduced rhamnosylation efficiency and accordingly all EF-P*_Ppu_* substitutions – except changes of R32 – were fully modified *in vivo* (Figure 3B). Regardless, S30P*_Ppu_* reaches only 4% of wild-type β-galactosidase activity and hence remains almost inactive even upon rhamnosylation (Figure 3B). This result is notably reminiscent of what we saw with the corresponding *E. coli* EF-P converse exchange P32S. Contrary to S30P*_Ppu_*, the alanine and glycine substitutions S30A*_Ppu_* and S30G*_Ppu_* reached 39 and 96% of wild-type β-galactosidase activity, respectively. These data support our observation of a strong selection against proline in the arginine-type EF-Ps, but at the same time allowing for a certain degree of freedom in the -2 position of the modification site. Substitutions of EF-P*_Ppu_* in positions N33, S34, and A35 as well as K29 are also tolerated without significant activity loss (Figure 3B). Similar to G33 in EF-P*_Eco_* (Yanagisawa et al., 2010; Figure 3A), the position equivalent G31 in EF-P*_Ppu_* is crucial for both modification efficiency and protein function (Figure 3B), which might be explained by sterically-hindering interactions with either the ribosome or EarP caused by longer side chains. Interestingly and in contrast to K34R*_Eco_*, R32K*_Ppu_* is not only inactive but the β-galactosidase activity measured with this variant is even below the level of an Δ*efp_Eco_* deletion strain. This drastic phenotype indicates an inhibitory effect on polyproline translation. Notably, we saw the same when testing unmodified EF-P of both *P. putida* (Figure 1D) or *S. oneidensis* (Lassak et al., 2015). A similar phenomenon was also observed by others when analyzing the growth of *P. aeruginosa* harboring the EF-P R32K variant (Rajkovic et al., 2015). The most plausible explanation is a distinct orientation of the protruding residue that depends on the β3Ωβ4 composition.

### The Essential Proline P32 in *E. coli* EF-P Prevents Activation With Rhamnosylarginine

**FIGURE 4:**
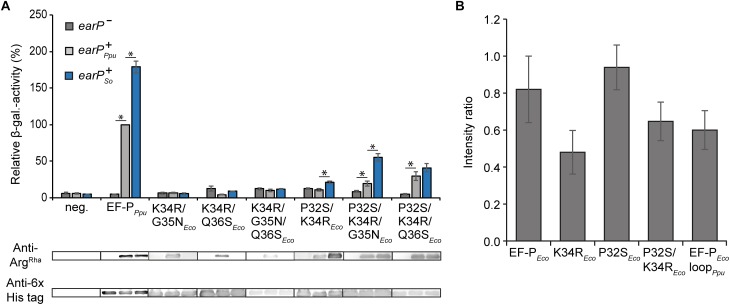
*In vivo* and *in vitro* characterization of EF-P hybrids **(A)** Top: *In vivo* rhamnosylation and functionality analysis of EF-P hybrids co-expressed with EarP from *P. putida* or *S. oneidensis*. Measurements were performed in the *E. coli* reporter strain (MG-CR-efp-KanS). *E. coli*Δ*efp* cells were complemented either with plasmid encoded His_6_-tagged EF-P*_Eco_*_/_*_Ppu_* variants solely (*earP^-^*, dark gray bars) or in combination with either EarP from *P. putida* (*earP*^+^*_Ppu_*, light gray bars) or EarP from *S. oneidensis* (*earP*^+^*_So_*, blue bars). β-galactosidase activities are given in relative MU with the wild-type EF-P*_Ppu_* in *earP*^+^_Ppu_ cells set to 100%. Asterisks indicate significant (*P* < 0.05) differences in the ribosome rescue activity of a given EF-P*_Eco_* variant in the presence of EarP*_Ppu_* or EarP*_So_* compared to the same variant in the absence of EarP. Bottom: *In vivo* rhamnosylation levels were detected and visualized using Anti-Arg^Rha^. Production of EF-P*_Ppu_* and EF-P*_Eco_* variants was detected and visualized using Anti-6×His^®^. **(B)** NMR-single titration experiment: Average change in the intensity ratio of EF-P*_Eco_* and its variants upon titration with twofold EarP*_Ppu_*. Error bars represent standard deviation of the intensity ratios over all signals within each titration.

Our mutational analysis clearly shows that the presence of P32 is crucial for the activity of EF-P*_Eco_* on the one hand and prevents ribosome rescue by EF-P*_Ppu_* on the other hand. Accordingly, we were curious whether a double substitution P32S/K34R*_Eco_* is sufficient to become translationally active upon modification by EarP (Figure 4A). Unfortunately, this EF-P variant was unable to promote CadC translation as seen by the low β-galactosidase activities in the Δ*efp_Eco_* P*_cadBA_*::*lacZ* reporter strain. However, at the same time we noticed a reduced *in vivo* rhamnosylation efficiency, which might mask a potential rescue effect. Therefore, we tested whether the EarP ortholog from *S. oneidensis* MR–1 might be a more efficient modifier. Indeed, upon co-expression of *earP_So_*, rhamnosylation of P32S/K34R*_Eco_* reached significantly higher levels, which concomitantly resulted in a partial ribosome rescue (Figure 4A). To understand the effect of substituting *E. coli* EF-P P32 especially on rhamnosylation efficiency by EarP*_Ppu_* we determined its interactions with wildtype EF-P*_Eco_* and variants at a molecular level by performing NMR titration experiments (Figure 4B and Supplementary Figures S5A,B). EF-P*_Eco_* interacts with EarP*_Ppu_* as shown by the decrease in total amount of peak intensities in the EF-P*_Eco_* ^15^N-HSQC spectrum upon EarP*_Ppu_* titration. Physical interaction leads to an increased molecular tumbling time, which in turn decreases transverse relaxation times and peak intensities. The interaction was substantially enhanced in the K34R*_Eco_* variant, resulting in even lower peak intensities. This was expected, as R34 makes important contacts with EarP and its cognate EF-P in *Neisseria meningitidis* (Sengoku et al., 2018). In contrast to K34R*_Eco_*, we observed reduced interaction strength in the P32S variant as peak intensities were stronger than for EF-P*_Eco_* wild type. This result might be counterintuitive, however, only if one ignores that EF-P must not only be efficiently rhamnosylated by EarP, but at the same time has to interact optimally with the P-site tRNA on the ribosome. In this light, the substitution of proline might be regarded as an evolutionary consequence to maintain functionality at the expense of rhamnosylation efficiency. In line with the findings for K34R*_Eco_* and P32S*_Eco_*, the K34R/P32S*_Eco_* double substitution variant showed intermediate interaction with EarP*_Ppu_* compared to K34R*_Eco_* and increased further with the EF-P*_Eco_* loop*_Ppu_* variant. In addition to K34R*_Eco_* and P32S*_Eco_*, the EF-P*_Eco_* loop*_Ppu_* construct bears two additional substitutions at positions 35 and 36, which seem to be important for EF-P/EarP interaction. Thus, we can interpret our finding as an adjustment to compensate for the negative interaction effect that we saw with P32S*_Eco_*.

It is possible that substitution of proline P32 causes substantial changes in the loop dynamics due to its rigid nature. To test this, we performed ^15^N *R*_1_, *R*_2_, and steady-state heteronuclear {^1^H}–^15^N-NOE relaxation experiments on EF-P*_Eco_* and its variants and compared it with EF-P*_Ppu_*. Our analysis suggests that substitution of single EF-P*_Eco_* β3Ωβ4-loop residues with residues from EF-P*_Ppu_* or even with the complete β3Ωβ4 does not significantly alter the NMR relaxation properties of β3Ωβ4 and hence its dynamics (Supplementary Figures S5C–F). Thus, differences observed in the interaction of EF-P*_Eco_* and its variants with EarP*_Ppu_* can be attributed to the molecular nature of resulting interactions rather than changes in the loop dynamics.

Our observation that substitution of P32 weakens the interaction strength between EF-P*_Eco_* and EarP*_Ppu_* explains the differences in cross-complementing the Δ*efp* P*_cadBA_*::*lacZ* mutant phenotype with K34R/P32S*_Eco_* in combination with the rhamnosyltransferase ortholog either from *S. oneidensis* or *P. putida* (Figure 4A). It is also indicative that further sequence determinants in the β3Ωβ4 are contributing to EF-P recognition by EarP and accordingly being in line with our *in vitro* rhamnosylation studies on EF-P*_Ppu_* substitution variants (Figure 3B). Consequently, we additionally substituted G35 and Q36 for asparagine and serine, respectively. Both resultant EF-P*_Eco_* variants P32S/K34R/G35N*_Eco_* and P32S/K34R/Q36S*_Eco_* alleviated CadC translation when co-producing EarP*_Ppu_*, exhibited by a twofold and threefold increase in β-galactosidase activities, respectively (Figure 4A). However, neither the double substitution K34R/G35N*_Eco_*, K34R/Q36S*_Eco_* nor the triple exchange K34R/G35N/Q36*_Eco_* had an alleviating effect on the translational arrest (Figure 4A). In summary, our analysis clearly shows that cross-activation of EF-P*_Eco_* by EarP*_Ppu_* is strictly prohibited in the presence of P32, whereas on the contrary cross-modification solely depends on the protruding residue to be arginine. Combined with our *in vitro* interaction analysis we conclude that specifically the selection against that proline is an adaptation to rescue polyproline stalled ribosomes with α-rhamnosylarginine rather than for efficient modification (Supplementary Figure S2B). On the other hand, our data also implies that additional adjustments in the β3Ωβ4 sequence composition have been made to compensate for the negative effect of rhamnosylation (Figure 1D).

### The Phylogeny of the β3Ωβ4 Composition Unveils the Functional Relationship Between P32 and the Modification Site

Our findings prompted us to investigate the evolutionary order of events resulting in the observed co-occurrence patterns between the residues occupying either the modification site (position 34 according to the numbering of *E. coli* EF-P) or the respective position two amino acids upstream. To this end, we performed a phylogenetic tree reconstruction using the maximum likelihood method from the phytools R package (Revell, 2012). We note that, according to the given bootstrap values, especially the deep branches of the tree might be rather randomly placed and thus the obtained results should be handled with caution (Supplementary Datasets S2, S3). Nevertheless, in combination with our biochemical and biophysical data these bioinformatics analyses might provide a plausible rationale for the β3Ωβ4 composition and EF-P modification strategies.

As the lysine at the β3Ωβ4 tip was found in more than three quarters of all EF-P sequences and is also conserved in the eukaryotic and archaeal orthologs e/aIF5A (Dever et al., 2014), we hypothesized that this amino acid is evolutionary ancient. Indeed, we found EF-P with a protruding lysine to be most likely at the root of our tree with subsequent emergence of the first arginine, followed by asparagine, glutamine, and methionine (Supplementary Figure S6A).

**FIGURE 5:**
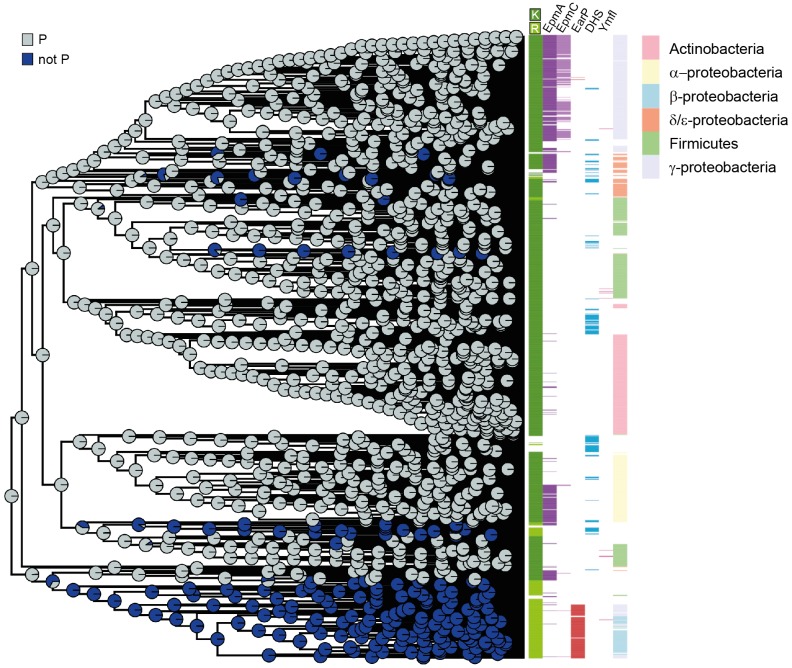
EF-P phylogeny and β3Ωβ4 sequence evolution **(A)** Phylogenetic tree reconstruction of the 32nd position (–2 relative to the β3Ωβ4 tip) of the EF-P KOW-like N-domain I. The colored circle areas correspond to the likelihood of the 32nd position to contain a proline (state “P”) or another amino acid residue (“not P”). **(B)** Bar colors indicate, from left to right, the amino acid located at the 34th position (β3Ωβ4 tip), the presence/absence of certain modification enzymes, and the taxonomy annotation for Proteobacteria, Firmicutes and Actinobacteria. Each colored line corresponds to one bacterial species in the EF-P data set (Supplementary Dataset S1).

When reconstructing evolutionary scenarios for position 32 (Figure 5A), proline is the most likely amino acid in an EF-P progenitor. The subsequent selection against it in certain EF-P subpopulations strongly correlates with arginine in the protruding position (Supplementary Figure S6A) and in turn strengthens our observations that this specific residue is crucial for optimal orientation of β3Ωβ4 (Figure 3, 4). This scenario is further corroborated by the fact that the structural restrictions caused by proline in position 32 have favored the evolution of lysine β-lysylation and lysine 5-aminopentanolylation. Whereas P32 seems to be incompatible with rhamnosylation. Our data further implies that the modification system for the latter emerged subsequent to the phylogenetic recruitment of R34/noP32 (Figure 5B and Supplementary Figure S6B).

## Conclusion

In this study we provide a comprehensive analysis of EF-P β3Ωβ4 and how its sequence composition allows functionalization by chemically and structurally distinct modifications. It might also help to predict the type of novel, yet undiscovered EF-P post-translational functionalization strategies in the >50% of bacteria which do not encode any known modification enzyme. Our assumption is supported by the recent identification of lysine 5-amino-pentanolylation which takes place in *B. subtilis* and presumably a few other firmicutes (Hummels et al., 2017). This EF-P activation strategy chemically resembles β-lysylation and also occurs on a β3/β4 loop with an invariant proline two amino acids upstream of the modification site. Further, in certain prokaryotes that have a β3Ωβ4 similar to EF-P*_Eco_* with lysine at the loop tip or alternatively an asparagine (Figure 2D), one can identify a deoxyhypusine synthase (DHS) like protein (Figure 5B; Brochier et al., 2004). In eukaryotes and archaea, DHS elongates a lysine in the EF-P ortholog IF5A by an amino-butyryl moiety (Wolff et al., 1990; Prunetti et al., 2016). Accordingly, it is plausible that the bacterial ortholog might attach an analogous modification onto the respective EF-Ps although the experimental connection remains elusive.

The evolutionary flexibility in modification systems and β3Ωβ4 sequence composition is not fully understood yet. However, one could speculate that besides the universally conserved role in alleviating ribosome stalling at polyproline stretches (Gutierrez et al., 2013; Ude et al., 2013) diverse EF-Ps might have extended functionality. In this regard it was reported (Pelechano and Alepuz, 2017; Schuller et al., 2017) that IF5A also acts on non-polyproline arrest motifs and even facilitates termination. Although EF-P activity seems to be restricted to the alleviation of translational arrest situations at consecutive prolines (Ude et al., 2013; Woolstenhulme et al., 2015) it should be noted that all global analyses thus far were performed solely in *E. coli* (Peil et al., 2013; Woolstenhulme et al., 2015) and *Salmonella enterica* (Hersch et al., 2013), both of which depend on (*R*)-β-lysylation of lysine. One might therefore speculate whether other EF-Ps with distinct modifications and β3Ωβ4 sequence composition might have expanded functions similar to eIF5A. EarP-dependent EF-Ps might therefore be of particular interest. First evolved in β-proteobacteria, this EF-P type seems to have spread into certain γ-proteobacterial orders and other phyla (Lassak et al., 2015). Conversely, however, horizontal gene transfer events of EpmABC-dependent EF-Ps into the β-proteobacterial subdivision hardly occur. This in turn could indicate a selection in favor of EF-P arginine rhamnosylation caused either by an expanded target spectrum or improved functionality.

The results of this study also demonstrate the possibility of switching the EarP acceptor substrate specificity. The interaction of EarP with its cognate EF-P has been shown to be both sequence- and structure dependent (Krafczyk et al., 2017; Sengoku et al., 2018). Our data show that a substitution of lysine to arginine in the EF-P of *E. coli* K34R*_Eco_* is already sufficient to allow for rhamnosylation in an EarP dependent manner. As the EF-Ps of *E. coli* and *P. putida* share only 30% identity in the EarP-interacting EF-P_N domain, sequence-specific contacts between EF-P and EarP (Krafczyk et al., 2017; Sengoku et al., 2018) might only enhance interaction strength between the two proteins. This is further supported by our corresponding bacterial two-hybrid and *in vitro* NMR analyses. The recognition motif for the AIDA-associated heptosyltransferase Aah has been described as a “short β-strand–short acceptor loop–short β-strand” (Charbonneau et al., 2012). Analogously the two beta-strands bracketing β3Ωβ4 might constitute a structural recognition motif for EarP dependent rhamnosylation. Determining the minimal recognition motif is of particular interest as this information allows for targeted rhamnosylation even for proteins other than EF-P. Thus, our study also lays the foundation to evolve EarP into a glycosynthase that can ultimately be used in heterologous production of eukaryotic glycoproteins.

## Author Contributions

DF, MPa, EM, and JL performed the bioinformatic analyses. JH, PJ, and JM performed the NMR studies. WV produced and purified the corresponding proteins. MPf performed isoelectric focusing experiments. RK and ZG performed *in vitro* rhamnosylation assays. WV and RK conducted all other biochemical and genetic analyses of β3Ωβ4 substitution variants of *E. coli* and *P. putida*. WV and MF performed the biochemical analysis with EarP from *S. oneidensis* with contributions from JL. JL, JH, KJ, and DF designed the study. WV, RK, KJ, EM, PJ, JH, and JL wrote the manuscript.

## Conflict of Interest Statement

The authors declare that the research was conducted in the absence of any commercial or financial relationships that could be construed as a potential conflict of interest.
