# Supplementary material for: Switching the Post-translational Modification of Translation Elongation Factor EF-P
Source: Front Microbiol. 2019 May 24;10:1148. doi: 10.3389/fmicb.2019.01148 (PMC6544042; doi:10.3389/fmicb.2019.01148)
Supplement: Supplementary file 2 [file Data_Sheet_2.pdf]

Supplementary Dataset S2: EF-P phylogenetic tree including bootstrap support values

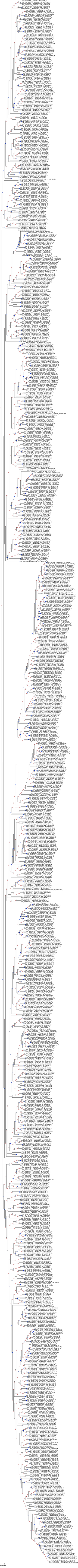
0.837875
