# Supplementary material for: Switching the Post-translational Modification of Translation Elongation Factor EF-P
Source: Front Microbiol. 2019 May 24;10:1148. doi: 10.3389/fmicb.2019.01148 (PMC6544042; doi:10.3389/fmicb.2019.01148)
Supplement: Supplementary file 4 [file Data_Sheet_4.pdf]

Supplementary Material

1 Supplementary Figures and Tables

1.1 Supplementary Figures

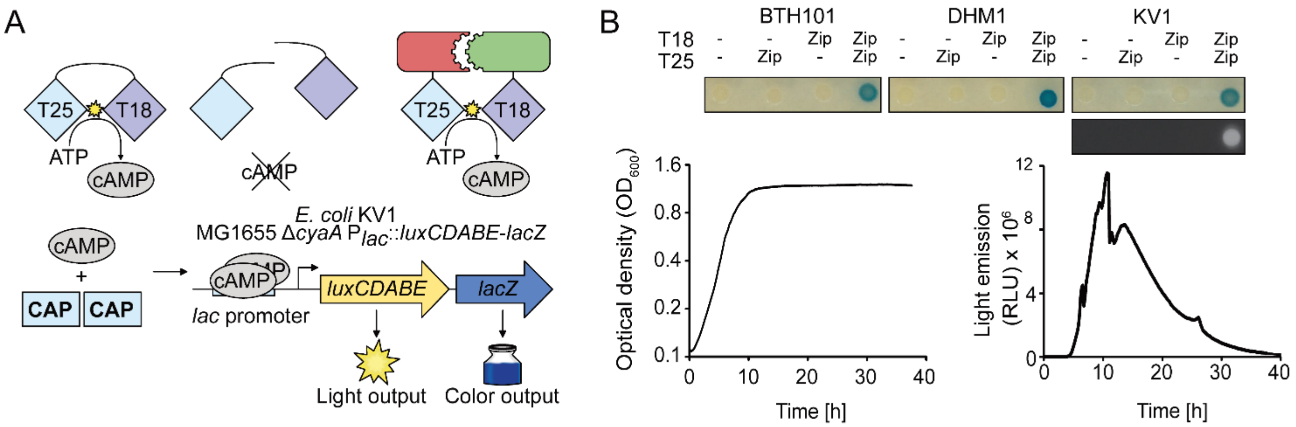

Figure S1. (A) Molecular principle of the Euromedex bacterial two-hybrid system with *E. coli* KV1 (adjusted from the Euromedex KIT Manual): The *Bordetella pertussis* adenylate cyclase CyaA apoprotein catalyzes the formation of cyclic AMP from ATP. Splitting the enzyme into two parts – T18 and T25 – renders CyaA inactive even upon co-expression. Fusing T18 and T25 to interacting proteins brings the fragments into close proximity and thus allows reconstitution of the Apo-CyaA. In *E. coli* KV1, the cyclic AMP dependent lac promoter precedes a translational fusion of the *lux*-operon and *lacZ*, allowing the indirect measurement of protein-protein interactions by light emission and colorimetric detection. **(B)** Proof of principle of *E. coli* KV1 as an *in vivo* reporter for protein-protein interaction using the self-interacting leucine zipper of GCN4. The colorimetric detection in *E. coli* KV1 cells was assessed semi quantitatively based on the formation of blue colonies on LB (Miller)-plates containing 40 µg/mL X-Gal. *E. coli* KV1 light emission was measured in a time course experiment recording relative luminescence (RLU) and optical density at 600 nm (OD600) in time intervals of 10 minutes.

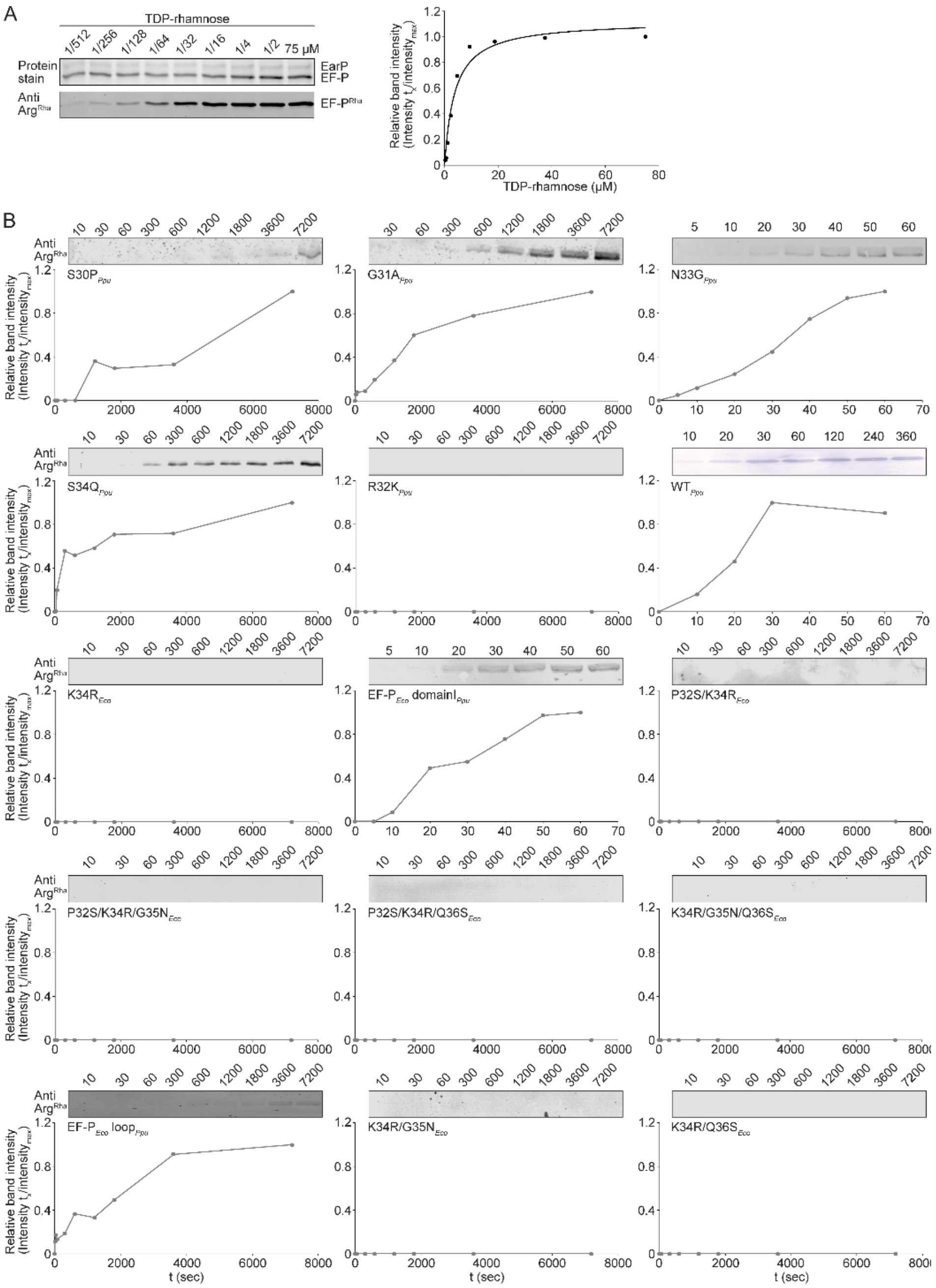

Figure S2. (A) Left: Analysis of EarP*Ppu* kinetic parameters. 0.5 µg of EF-P*Ppu* and 0.05 µg of EarP*Ppu* were subjected to SDS-PAGE after *in vitro* rhamnosylation (see Material and Methods) for 20 seconds at varying TDP-rhamnose concentrations. Proteins were transferred to nitrocellulose membrane by horizontal Western blotting. Rhamnosylated EF-P*Ppu* was detected using 0.25 µg/ml of Anti-Arg^{Rha}. Right: TDP-rhamnose saturation curve of EarP*Ppu*. Band intensities on nitrocellulose membrane were quantified using ImageJ and relative band intensities were plotted against TDP-rhamnose concentration. **(B)** Timecourse analysis of various EF-P*Ppu* and EF-P*Eco* variants. 0.5 µg of EF-P*Ppu* and 0.05 µg of EarP*Ppu* were subjected to SDS-PAGE after *in vitro* rhamnosylation at a TDP-rhamnose concentration of 50 µM for varying timespans. Band intensities on nitrocellulose membrane were quantified using ImageJ and relative band intensities were plotted against time.

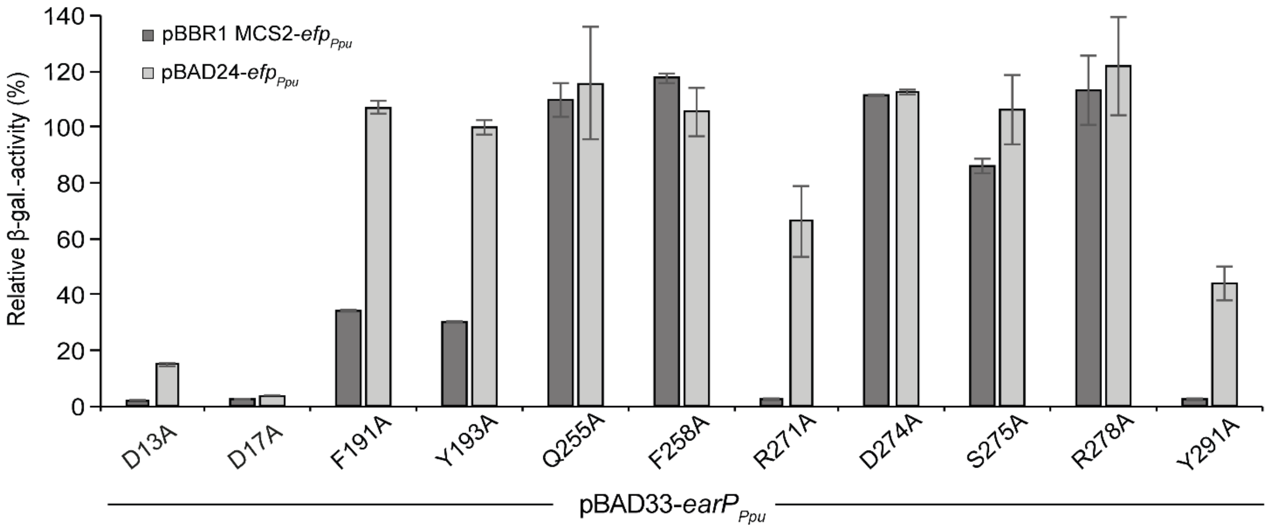

Figure S3. β-galactosidase activity in *E. coli* MG-CR-*efp*-Kan^{S} upon expression (dark grey) or co-overexpression (light grey) of EF-P*Ppu* and EarP*Ppu* and single-amino-acid substitution variants. Cells were incubated under *cadBA*-inducing conditions (LB, pH 5.8) at 30°C o/n.

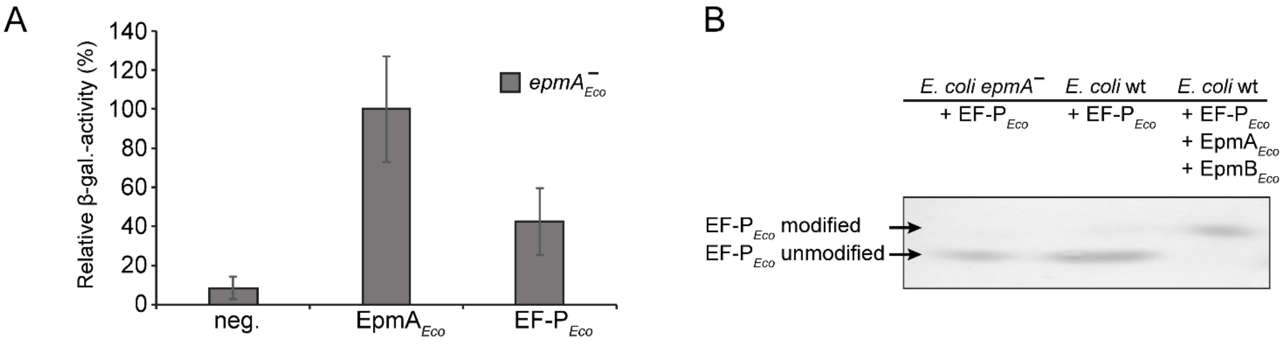

Figure S4. **(A)** β-galactosidase activity in the Δ*epmA* reporter strain (*E. coli* MG-CL-12-yjeA) harboring either a plasmid borne copy of *epmA* (pBAD33-*epmA*) or *efp* (pBAD24-*efpEco*). **(B)** Isoelectric focusing of *E. coli* EF-P, either overproduced in *E. coli epmA*$^{-}$ (BW25113-epmA), in which *epmA* was chromosomally deleted, or in *E. coli* wild type cells (BW25113). Furthermore, EF-P was overproduced in combination with its PTM proteins EpmA and EpmB in *E. coli* wild type (BW25113). Production of EF-P was verified by Western blot analysis.

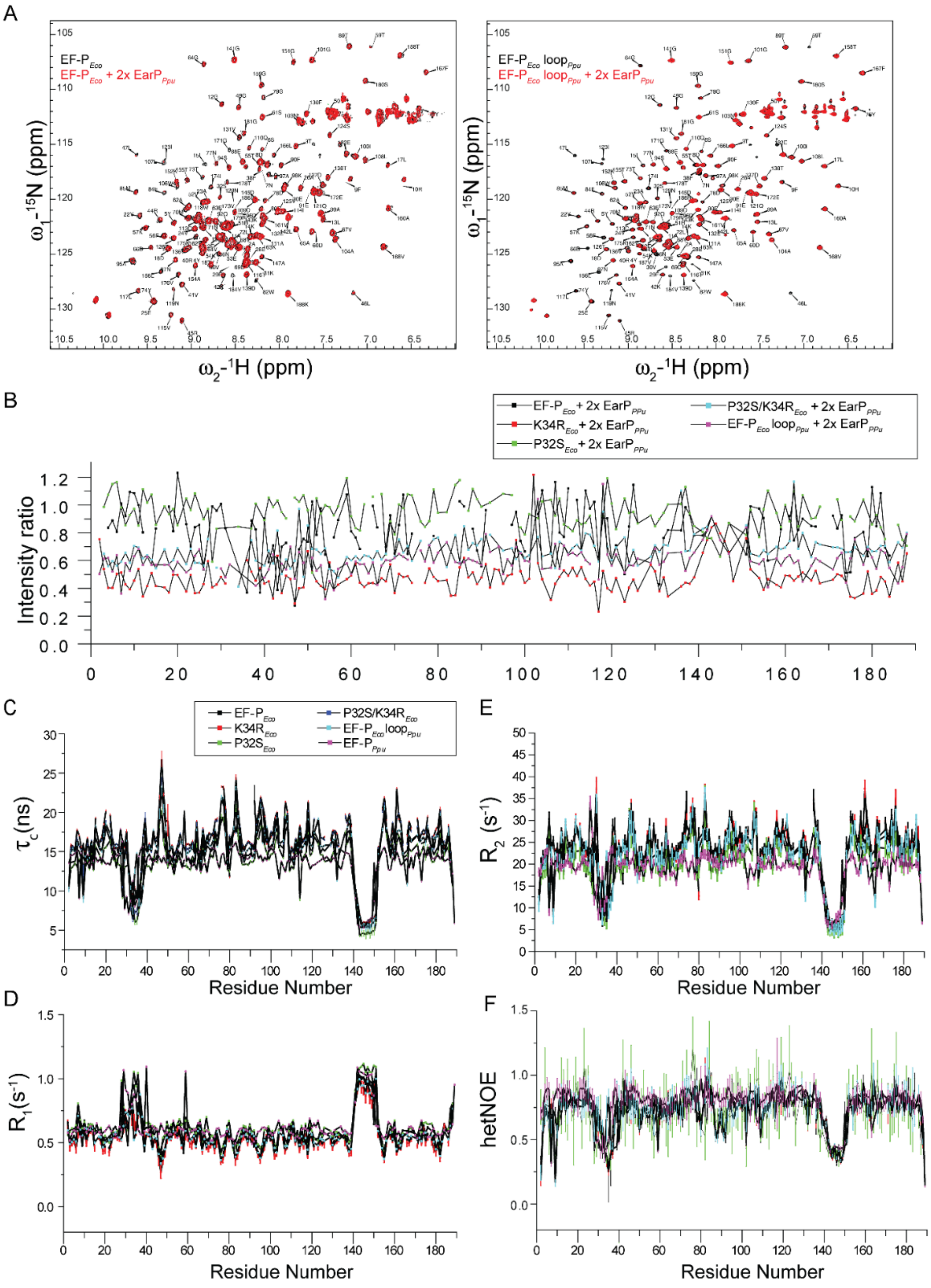

Figure S5. **(A)**^{1}H-^{15}N HSQC titrations of EF-P$_{Eco}$ and EF-P$_{Eco}$ loop$_{Ppu}$ with EarP$_{Ppu}$ is shown along with the backbone assignments. Both proteins show a decrease in intensity upon titration with EarP$_{Ppu}$ indicating their interaction with EarP$_{Ppu}$. **(B)** Intensity ratio of all the EF-P$_{Eco}$ mutants on titration with EarP$_{Ppu}$ is shown. **(C)** The correlation time (τ_c) for EF-P$_{Eco}$ and its variants along with **(D)** R1, **(E)** R2 rates and **(F)** hetNOE are shown.

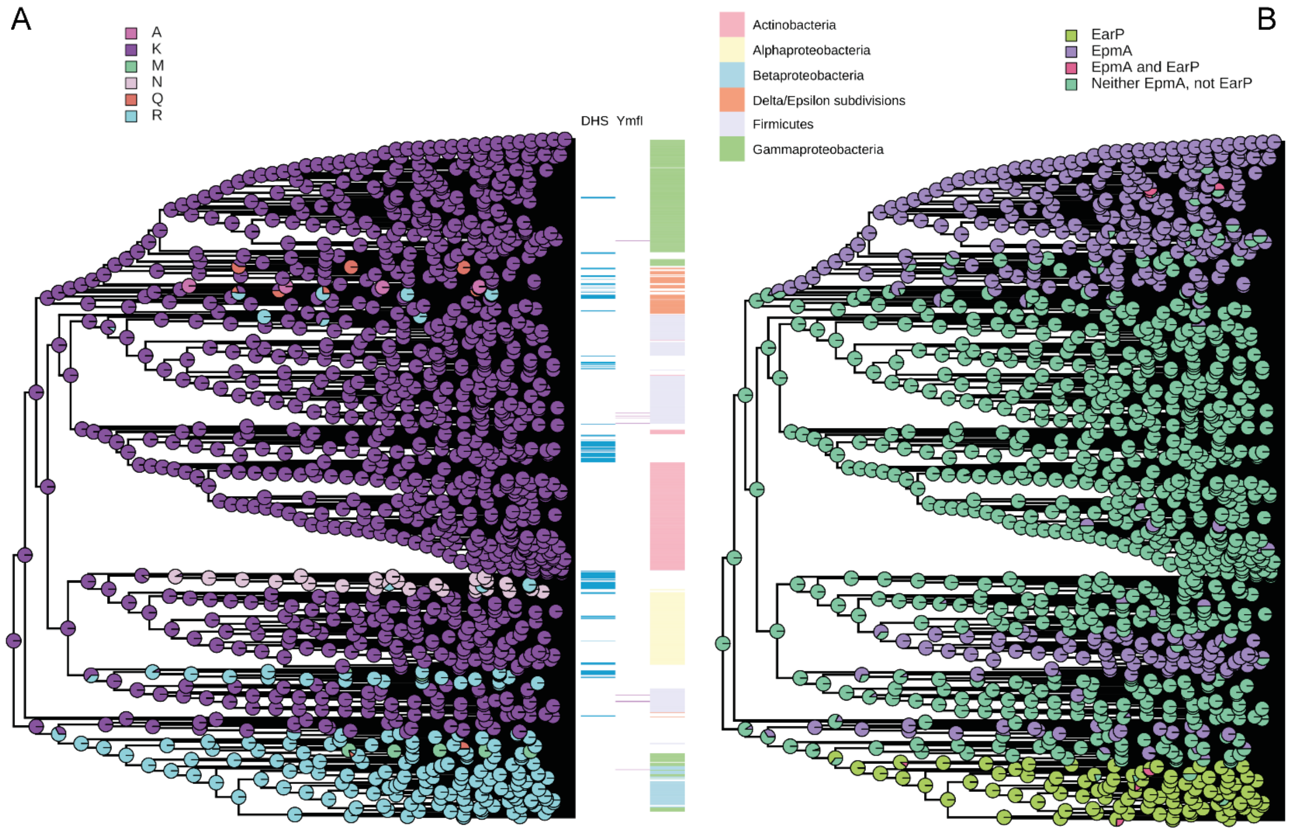

Figure S6. **(A)** Phylogenetic trees of the EF-P KOW-like N-domain I, with reconstructed state of the 34th position (A, left), presence of known modification systems and taxonomy (A, right colored bars). **(B)** Phylogenetic reconstruction of emergence of EF-P modification systems.

1.2 Supplementary Tables

Table S1. Strains used in this study

Strain	Genotype	Reference
DH5α*λpir*	*recA1 endA1 gyrA96 thi-1 hsdR17 supE44 relA1* Δ*lacZYA-argF* U169 ϕ80d*lacZ*ΔM15 λpir	(Macinga et al., 1995)
LMG194	F^- Δ*lacX74 galE galK thi rpsL* Δ*phoA* (PvuII) Δ*ara714 leu*::Tn*10*	(Guzman et al., 1995)
BL21(DE3)	F^- *ompT gal dcm lon* $hsdS_B$(rB^- mB^-) λ(DE3)	(Studier and Moffatt, 1986)
DHM1	F^- *cya-854 recA1 endA1 gyrA96* (*NalR*) *thi1 hsdR17 spoT1 rfbD1 glnV44*(*AS*)	(Karimova et al., 2005)
BTH101	F^- *cya-99 araD139 galE15 galK16 rpsL1* (*StrR*) *hsdR2 mcrA1 mcrB1* additional *relA1* mutation reported by (Battesti and Bouveret, 2012)	Euromedex
JW4107	Δ*(araD-araB)567*, Δ*lacZ4787*(::rrnB-3), λ^-, *rph-1*, Δ*(rhaD-rhaB)568*, Δ*efp-772::kan*, *hsdR514*	(Baba et al., 2006)
KV1	MG1655 *rpsL150* Δ*cyaA* P_{lac}::*luxCDABE-lacZ*	This study
LF1	MG1655 *rpsL150* P_{lac}*::rpsL-neo-kan::lacZ*$^{\Delta 1\text{–}100\ bp}$*;Kan*^{R} *Strp*^{S}	(Fried et al., 2012)
MG-CR-*efp*	MG1655 Δ*lacZ*::*tet rpsL*150 *efp*::*npt* Δ*cadBA* P_{cadBA}::*lacZ*	(Lassak et al., 2015)
MG-CR-*efp*-KanS	MG1655 Δ*lacZ*::*tet rpsL*150 Δ*efp* Δ*cadBA* P_{cadBA}::*lacZ*	This study
MG-CR-*efp-epmA*-KanR	MG1655 Δ*lacZ*::*tet rpsL*150 Δ*efp* Δ*cadBA epmA*::*npt* P_{cadBA}::*lacZ*	This study
MG-CL-12-*yjeA*	MG1655 Δ*lacZ*::*tet rpsL*150 *yjeA*::*npt* Δ*cadBA cadBA*::*lacZ*	(Ude, 2013)
BW25113	Δ(*araD-araB*)*567*, Δ*lacZ4787*(::rrnB-3), λ-, rph-1, Δ(*rhaD-rhaB*)*568*, *hsdR514*	(Datsenko and Wanner, 2000)
BW25113-*epmA*	Δ(araD-araB)567, ΔlacZ4787(::rrnB-3), λ-, rph-1, Δ(rhaD-rhaB)568, hsdR514, ΔepmA	This study
JW4116	F^-, *Δ(araD-araB)567*, *ΔlacZ4787*(::rrnB-3), λ^-, *rph-1*, *Δ(rhaD-rhaB)568*, *ΔpoxA782::kan*, *hsdR514*	(Baba et al., 2006)

Kan^R: kanamycin resistance, $Srep^S$: streptomycin sensitive

Table S2. Plasmids used in this study

**Plasmid**	**Features**	**Reference**
**Plasmids for strain construction**		
pRED/ET® Amp	λ-RED recombinase in pBAD24; Amp^R	GeneBridges, Germany
FRT-PGK-gb2-neo-FRT template DNA	PCR-template (plasmid DNA) for generating a FRT-flanked PGK-gb2-neo cassette, Kan^R	GeneBridges, Germany
709-FLPe, amp	Plasmid for removal of FRT flanked resistance cassette, Supplier ID: A106	GeneBridges, Germany
pBAD/HisA-Lux	Contains the *luxCDABE* operon from *Photorhabdus luminescens*	(Volkwein et al., 2017)
**Plasmids for mutational analysis of the *E. coli* loop and for overproduction**		
pBAD24	Amp^R-cassette, pBBR322 origin, *araC* coding sequence, *ara* operator	(Guzman et al., 1995)
pBAD24-efp_{Eco}	C-terminal His_6-tagged *E. coli efp* amplified from pBAD33-$efp_{E.c.}$-His6 (Lassak et al., 2015) using P1/P2	this study
pBAD24-efp_{Eco} P32G	C-terminal His_6-tagged *E. coli efp* substitution variant P32G. Overlap PCR fragment was amplified from pBAD24-efp_{Eco} using P1/P16 and P2/P15	this study
pBAD24-efp_{Eco} P32S	C-terminal His_6-tagged *E. coli efp* substitution variant P32S. Overlap PCR fragment was amplified from pBAD24-efp_{Eco} using P1/P18 and P2/P17	this study
pBAD24-efp_{Eco} G33A	C-terminal His_6-tagged *E. coli efp* substitution variant G33A. Overlap PCR fragment was amplified from pBAD24-efp_{Eco} using P1/P20 and P2/P19	this study
pBAD24-efp_{Eco} G33S	C-terminal His_6-tagged *E. coli efp* substitution variant G33S. Overlap PCR fragment was amplified from pBAD24-efp_{Eco} using P1/P22 and P2/P21	this study
pBAD24-efp_{Eco} K34A	C-terminal His_6-tagged *E. coli efp* substitution variant K34A. Amplified from pBAD33-$efp_{E.c.}$-His6-K34A (Lassak et al., 2015) using P1/P2	this study
pBAD24-efp_{Eco} K34M	C-terminal His_6-tagged *E. coli efp* substitution variant K34M. Overlap PCR fragment was amplified from pBAD24-efpEco using P1/P24 and P2/P23	this study

pBAD24-*efp*$_{Eco}$ K34N	C-terminal His$_6$-tagged *E. coli efp* substitution variant K34N. Overlap PCR fragment was amplified from pBAD24-*efp*$_{Eco}$ using P1/P26 and P2/P25	this study
pBAD24-*efp*$_{Eco}$ K34Q	C-terminal His$_6$-tagged *E. coli efp* substitution variant K34Q. Overlap PCR fragment was amplified from pBAD24-*efp*$_{Eco}$ using P1/P28 and P2/P27	this study
pBAD24-*efp*$_{Eco}$ K34R	C-terminal His$_6$-tagged *E. coli efp* substitution variant K34R. Amplified from pBAD33-*efp*$_{E.c.}$-His6-K34R (Lassak et al., 2015) using P1/P2	this study
pBAD24-*efp*$_{Eco}$ G35N	C-terminal His$_6$-tagged *E. coli efp* substitution variant G35N. Overlap PCR fragment was amplified from pBAD24-*efp*$_{Eco}$ using P1/P30 and P2/P29	this study
pBAD24-*efp*$_{Eco}$ Q36S	C-terminal His$_6$-tagged *E. coli efp* substitution variant Q36S. Overlap PCR fragment was amplified from pBAD24-*efp*$_{Eco}$ using P1/P32 and P2/P31	this study
**Plasmids for mutational analysis of the *P. putida* loop and for overproduction**		
pBAD24-*efp*$_{Ppu}$	C-terminal His$_6$-tagged *P. putida efp* amplified from *Pseudomonas putida* KT2440 using P1/P2	this study
pBAD24-*efp*$_{Ppu}$ K29A	C-terminal His$_6$-tagged *efp P. putida* substitution variant K29A. Overlap PCR fragment was amplified from pBAD24-*efp*$_{Ppu}$ using P1/P36 and P2/P35	this study
pBAD24-*efp*$_{Ppu}$ K29R	C-terminal His$_6$-tagged *P. putida efp* substitution variant K29R. Overlap PCR fragment was amplified from pBAD24-*efp*$_{Ppu}$ using P1/P37 and P2/P35	this study
pBAD24-*efp*$_{Ppu}$ S30A	C-terminal His$_6$-tagged *P. putida efp* substitution variant S30A. Overlap PCR fragment was amplified from pBAD24-*efp*$_{Ppu}$ using P1/P38 and P2/P35	this study
pBAD24-*efp*$_{Ppu}$ S30G	C-terminal His$_6$-tagged *P. putida efp* substitution variant S30G. Overlap PCR fragment was amplified from pBAD24-*efp*$_{Ppu}$ using P1/P39 and P2/P35	this study
pBAD24-*efp*$_{Ppu}$ S30P	C-terminal His$_6$-tagged *P. putida efp* substitution variant S30P. Overlap PCR fragment was amplified from pBAD24-*efp*$_{Ppu}$ using P1/P40 and P2/P35	this study

pBAD24-*efp*$_{Ppu}$ G31A	C-terminal His$_6$-tagged *P. putida efp* substitution variant G31A. Overlap PCR fragment was amplified from pBAD24-*efp*$_{Ppu}$ using P1/P41 and P2/P35	this study
pBAD24-*efp*$_{Ppu}$ G31S	C-terminal His$_6$-tagged *P. putida efp* substitution variant G31S. Overlap PCR fragment was amplified from pBAD24-*efp*$_{Ppu}$ using P1/P42 and P2/P35	this study
pBAD24-*efp*$_{Ppu}$ R32K	C-terminal His$_6$-tagged *P. putida efp* substitution variant R32K. Overlap PCR fragment was amplified from pBAD24-*efp*$_{Ppu}$ using P1/P44 and P2/P43	this study
pBAD24-*efp*$_{Ppu}$ N33D	C-terminal His$_6$-tagged *P. putida efp* substitution variant N33D. Overlap PCR fragment was amplified from pBAD24-*efp*$_{Ppu}$ using P1/P46 and P2/P45	this study
pBAD24-*efp*$_{Ppu}$ N33G	C-terminal His$_6$-tagged *P. putida efp* substitution variant N33G. Overlap PCR fragment was amplified from pBAD24-*efp*$_{Ppu}$ using P1/P47 and P2/P45	this study
pBAD24-*efp*$_{Ppu}$ S34A	C-terminal His$_6$-tagged *P. putida efp* substitution variant S34A. Overlap PCR fragment was amplified from pBAD24-*efp*$_{Ppu}$ using P1/P48 and P2/P45	this study
pBAD24-*efp*$_{Ppu}$ S34Q	C-terminal His$_6$-tagged *P. putida efp* substitution variant S34Q. Overlap PCR fragment was amplified from pBAD24-*efp*$_{Ppu}$ using P1/P49 and P2/P45	this study
pBAD24-*efp*$_{Ppu}$ A35S	C-terminal His$_6$-tagged *P. putida efp* substitution variant A35S. Overlap PCR fragment was amplified from pBAD24-*efp*$_{Ppu}$ using P1/P50 and P2/P45	this study
pBAD33	Cm^R-cassette, p15A origin, *araC* coding sequence, *ara* operator	(Guzman et al., 1995)
pBAD33 PP1857-His6	C-terminal His6-Tag *earP* version from *P. putida* KT2440	(Krafczyk et al., 2017)

**Plasmids for cross modification/actication and overproduction**		
pBBRMCS2	pBBR origin of replication, *oriT*, KanR	(Kovach et al., 1995)
pBAD24-efp_{Eco} domainI-efp_{Ppu}	C-terminal His_6-tagged *E. coli efp* where the first 65 amino acids (domainI) were substituted by the first 65 amino acids from *P. putida efp*. Overlap PCR fragment was amplified from pBAD24-efp_{Ppu} using P1/P52 and pBAD24-efp_{Eco} P2/P51	this study
pBAD24-efp_{Eco} loop-efp_{Ppu}	C-terminal His_6-tagged *E. coli efp* P32S K34R G35N Q36S multiple amino acid substitution variant, corresponding to EF-P of *E. coli* carrying the acceptor loop of EF-P from *P. putida.* Overlap PCR fragment was amplified from pBAD24-efp_{Eco} using P1/P54 and P2/P53	this study
pBAD24-efp_{Eco} P32S K34R	C-terminal His_6-tagged *E. coli efp* substitution variant P32S K34R. Overlap PCR fragment was amplified from pBAD24-efp_{Eco} K34R using P1/P56 and P2/P55	this study
pBAD24-efp_{Eco} K34R G35N	C-terminal His_6-tagged *E. coli efp* substitution variant K34R G35N. Overlap PCR fragment was amplified from pBAD24-efp_{Eco} K34R using P1/P58 and P2/P57	this study
pBAD24-efp_{Eco} K34R Q36S	C-terminal His_6-tagged *E. coli efp* substitution variant K34R Q36S. Overlap PCR fragment was amplified from pBAD24-efp_{Eco} K34R using P1/P60 and P2/P59	this study
pBAD24-efp_{Eco} P32S K34R G35N	C-terminal His_6-tagged *E. coli efp* substitution variant P32S K34R G35N. Overlap PCR fragment was amplified from pBAD24-efp_{Eco} P32S K34R using P1/P62 and P2/P61	this study
pBAD24 -efp_{Eco} P32S K34R Q36S	C-terminal His_6-tagged *E. coli efp* substitution variant P32S K34R Q36S. Overlap PCR fragment was amplified from pBAD24-efp_{Eco} P32S K34R using P1/P64 and P2/P63	this study
pBAD24-efp_{Eco} K34R G35N Q36S	C-terminal His_6-tagged *E. coli efp* substitution variant K34R G35N Q36S. Overlap PCR fragment was amplified from pBAD24-efp_{Eco} K34R using P1/P66 and P2/P65	this study

pBAD33-*earP*$_{So}$	*earP* from *Shewanella oneidensis* MR-1 amplified from pBAD24-*earP*$_{S.o.}$-His$_6$ (Lassak et al., 2015) using P1/P67	this study
**Plasmids for XPPX assay**		
p3LC-TL30-APP	p3LC-TL30 + sequence encoding Ala-Pro-Pro	(Peil et al., 2013)
p3LC-TL30-PPD	p3LC-TL30 + sequence encoding Pro-Pro-Asp	(Peil et al., 2013)
p3LC-TL30-PPP	p3LC-TL30 + sequence encoding Pro-Pro-Pro	(Ude et al., 2013)
p3LC-TL30-DPP	p3LC-TL30 + sequence encoding Asp-Pro-Pro	(Peil et al., 2013)
p3LC-TL30-PPG	p3LC-TL30 + sequence encoding Pro-Pro-Gly	(Peil et al., 2013)
p3LC-TL30-PPN	p3LC-TL30 + sequence encoding Pro-Pro-Asn	(Peil et al., 2013)
**Plasmids used in the reporter strain MG-CL-12-yjeA**		
pBAD33-*epmA*	*epmA* from *E. coli*, amplified using P72/P75	this study
**Plasmids for protein overproduction (NMR)**		
pET SUMO	pBR322 origin, *lacI*, T7 *lac* promoter, N-terminal His$_6$ tag, SUMO coding sequence, Kan^R, Supplier ID: K300-01	Invitrogen
pET SUMO-*efp*$_{Eco}$	C-terminal genetic fusion of *efp* from *E. coli* to His$_6$-SUMO-tag. Amplified from pBAD24-*efp*$_{Eco}$ using P68/P69	this study
pET SUMO-*efp*$_{Ppu}$	C-terminal genetic fusion of *efp* from *P. putida* KT2440 to His$_6$-SUMO-tag	(Krafczyk et al., 2017)
pET SUMO-*efp*$_{Eco}$ P32S	C-terminal genetic fusion of *efp* from *E. coli* substitution variant P32S to His$_6$-SUMO-tag. Overlap PCR fragment was amplified from pET SUMO-*efp*$_{Eco}$ using P5/P18 and P6/P17	this study
pET SUMO-*efp*$_{Eco}$ K34R	C-terminal genetic fusion of *efp* from *E. coli* substitution variant K34R to His$_6$-SUMO-tag. Overlap PCR fragment was amplified from pET SUMO-*efp*$_{Eco}$ using P5/P71 and P6/P70	this study
pET SUMO-*efp*$_{Eco}$ P32S K34R	C-terminal genetic fusion of *efp* from *E. coli* substitution variant P32S K34R to His$_6$-SUMO-tag. Overlap PCR fragment was amplified from pET SUMO-*efp*$_{Eco}$ using P5/P56 and P6/P55	this study
pET SUMO-*efp*$_{Eco}$ loop$_{Ppu}$	C-terminal genetic fusion of *efp* from *E. coli* substitution variant P32S K34R G35N Q36S to His$_6$-SUMO-tag. Overlap PCR fragment was amplified from pET SUMO-*efp*$_{Eco}$ using P5/P54 and P6/P53	this study

**Plasmids for bacterial two-hybrid (BTH)**		
pUT18-*zip*	N-terminal genetic fusion of the leucine zipper from GCN4 to the T18 fragment of CyaA	Euromedex
pKT25-*zip*	C-terminal genetic fusion of the leucine zipper from GCN4 to the T25 fragment of CyaA	Euromedex
pUT18C-PP1858	C-terminal genetic fusion of *efp* from *Pseudomonas putida t*o the T18 fragment of CyaA	(Krafczyk et al., 2017)
pKT25-PP1857	C-terminal genetic fusion of *earP* from *Pseudomonas putida* to the T25 fragment of CyaA	(Krafczyk et al., 2017)
pUT18C-efp_{Eco}	C-terminal genetic fusion of *efp* from *Escherichia coli* to the T18 fragment of CyaA. PCR fragment was amplified from pBAD24-efp_{Eco} using P80/P81	this study
pUT18C-efp_{Eco} K34R	C-terminal genetic fusion of *efp* from *Escherichia coli* to the T18 fragment of CyaA. K34R single amino acid exchange variant. PCR fragment was amplified from pBAD24-efp_{Eco} K34R using P80/P81	this study
pUT18C-efp_{Eco} P32S K34R	C-terminal genetic fusion of *efp* from *Escherichia coli* to the T18 fragment of CyaA. P32S K34R double amino acid exchange variant. PCR fragment was amplified from pBAD24-efp_{Eco} P32S K34R using P80/P81	this study
pUT18C-efp_{Eco} $loop_{Ppu}$	C-terminal genetic fusion of *efp* from *E. coli* to the T18 fragment of CyaA. P32S K34R G35N Q36S multiple amino acid exchange variant, corresponding to EF-P of *E. coli* carrying the acceptor loop of EF-P from *P. putida.* PCR fragment was amplified from pBAD24-efp_{Eco} $loop_{Ppu}$ using P80/P81	this study
**Plasmids for isoelectric focusing**		
pBAD33-*efp*-His_6	C-terminal His_6-Tag *efp* version from *E. coli* into pBAD33	(Lassak et al., 2015)
pBAD33-*efp*-His_6-*epmAB*	C-terminal His_6-tagged *efp* and *epmAB from E. coli.* Overlap PCR fragment was amplified using P73/P76, P72/P75 and P74/P77	this study

Plasmids for EarP mutant test		
pBBR1-MCS2 NP_SO_PP1858-His6	C-terminal His6-Tag *efp* from *Pseudomonas putida* under control of the *Shewanella oneidensis* native *efp* promoter in pBBR1-MCS2	(Krafczyk et al., 2017)
pBAD33 PP1857 D13A-His6	C-terminal His_6-Tag *earP* from *Pseudomonas putida* in pBAD33. Mutant version; Asp13 exchanged to Ala	(Krafczyk et al., 2017)
pBAD33 PP1857 D17A-His6	C-terminal His_6-Tag *earP* from *Pseudomonas putida* in pBAD33. Mutant version; Asp17 exchanged to Ala	(Krafczyk et al., 2017)
pBAD33 PP1857 F191A-His6	C-terminal His_6-Tag *earP* from *Pseudomonas putida* in pBAD33. Mutant version; Phe191 exchanged to Ala	(Krafczyk et al., 2017)
pBAD33 PP1857 Y193A-His6	C-terminal His_6-Tag *earP* from *Pseudomonas putida* in pBAD33. Mutant version; Tyr193 exchanged to Ala	(Krafczyk et al., 2017)
pBAD33 PP1857 Q255A-His6	C-terminal His_6-Tag *earP* from *Pseudomonas putida* in pBAD33. Mutant version; Gln255 exchanged to Ala	(Krafczyk et al., 2017)
pBAD33 PP1857 F258A-His6	C-terminal His_6-Tag *earP* from *Pseudomonas putida* in pBAD33. Mutant version; Phe258 exchanged to Ala	(Krafczyk et al., 2017)
pBAD33 PP1857 R271A-His6	C-terminal His_6-Tag *earP* from *Pseudomonas putida* in pBAD33. Mutant version; Arg271 exchanged to Ala	(Krafczyk et al., 2017)
pBAD33 PP1857 D274A-His6	C-terminal His_6-Tag *earP* from *Pseudomonas putida* in pBAD33. Mutant version; Asp274 exchanged to Ala	(Krafczyk et al., 2017)
pBAD33 PP1857 S275A-His6	C-terminal His_6-Tag *earP* from *Pseudomonas putida* in pBAD33. Mutant version; Ser275 exchanged to Ala	(Krafczyk et al., 2017)
pBAD33 PP1857 R278A-His6	C-terminal His_6-Tag *earP* from *Pseudomonas putida* in pBAD33. Mutant version; Arg278 exchanged to Ala	(Krafczyk et al., 2017)
pBAD33 PP1857 Y291A-His6	C-terminal His_6-Tag *earP* from *Pseudomonas putida* in pBAD33. Mutant version; Tyr291 exchanged to Ala	(Krafczyk et al., 2017)

Amp^R, Cm^R, Kan^R: ampicillin, chloramphenicol, kanamycin resistance.

Table S3. Primers used in this study

Identifier	Oligonucleotide	Sequence (5' - 3')	Restriction site	Reference
**Primers for sequencing and cloning**				
P1	Seq33 fw	GGC GTC CAC ACT TTG CTA TGC		(Lassak et al., 2010)
P2	pBAD HisA rev	CAG TTC CCT ACT CTC GCA TG		(Lassak et al., 2010)
P3	*epmA* chk fw	TAG GTA CAA CAG TAT AGT CTG ATG GAT AA		this study
P4	*epmA* chk rev	TGA GGC ATG AAA CCA TCC TTC ATT TC		this study
P5	T7 Prom	TAA TAC GAC TCA CTA TAG G		
P6	T7 Term	TAT GCT AGT TAT TGC TCA G		
**Primers for strain construction of KV1**				
P7	*lacI*-583-fw	GTC TGC GTC TGG CTG GCT GGC ATA		(Fried et al., 2012)
P8	*luxC*-OL-rev	TAG TGC CCA TAG CTG TTT CCT GTG TGA AAT TGT TAT CC		this study
P9	*luxC*-OL-fw	GGA AAC AGC TAT GGG CAC TAA AAA AAT TTC ATT CAT TAT TAA CGG		this study
P10	*luxE*-OL-sRBS-*lacZ*-rev	AAT GTA CCT CCT TAC TTT ATT TAT TGT ATT TGT TTA GCT ATC AAA CGC TTC GGT TAA GCT C		this study
P11	OL-sRBS-*lacZ*-fw	ACA AAT ACA ATA AAT AAA GTA AGG AGG TAC ATT ATG ACC ATG ATT ACG GAT TCA CTG GCC G		this study
P12	*lacZ* 500bp anti	CGA CTG TCC TGG CCG TAA CCG ACC		(Fried et al., 2012)
P13	delta *cyaA* fw	GTT GGC GGA ATC ACA GTC ATG ACG GGT AGC AAA TCA GGC GAT ACG TCT TGA ATT AAC CCT CAC TAA AGG GCG		this study

P14	delta *cyaA* rev	TCC GCT AAG ATT GCA TGC CGG ATA AGC CTC GCT TTC CGG CAC GTT CAT CAT AAT ACG ACT CAC TAT AGG GCT C		this study
**Primers *E. coli* loop mutation and overproduction constructs**				
P15	*efp Eco* P32G fw	GTA AAA GGC GGT AAA GGC CAG G		this study
P16	*efp Eco* P32G rev	CCT GGC CTT TAC CGC CTT TTA C		this study
P17	*efp Eco* P32S fw	CGT AAA ATC GGG TAA AGG CCA GG		this study
P18	*efp Eco* P32S rev	CCT GGC CTT TAC CCG ATT TTA CG		this study
P19	*efp Eco* G33A fw	TAA AAC CGG CGA AAG GCC AGG		this study
P20	*efp Eco* G33A rev	CCT GGC CTT TCG CCG GTT TTA		this study
P21	*efp Eco* G33S fw	GTA AAA CCG TCG AAA GGC CAG G		this study
P22	*efp Eco* G33S rev	CCT GGC CTT TCG ACG GTT TTA C		this study
P23	*efp Eco* K34M fw	GTA AAA CCG GGT ATG GGC CAG GCA TTT		this study
P24	*efp Eco* K34M rev	AAA TGC CTG GCC CAT ACC CGG TTT TAC		this study
P25	*efp Eco* K34N fw	GTA AAA CCG GGT AAC GGC CAG GCA TTT		this study
P26	*efp Eco* K34N rev	AAA TGC CTG GCC GTT ACC CGG TTT TAC		this study
P27	*efp Eco* K34Q fw	GTA AAA CCG GGT CAG GGC CAG GCA TTT		this study
P28	*efp Eco* K34Q rev	AAA TGC CTG GCC CTG ACC CGG TTT TAC		this study
P29	*efp Eco* G35N fw	CCG GGT AAA AAC CAG GCA TTT GC		this study
P30	*efp Eco* G35N rev	GCA AAT GCC TGG TTT TTA CCC GG		this study
P31	*efp Eco* Q36S fw	GGG TAA AGG CAG CGC ATT TGC		this study

P32	*efp Eco* Q36S rev	GCA AAT GCG CTG CCT TTA CCC		this study
**Primers *P. putida* loop mutation and overproduction constructs**				
P33	NheI-NRBS-PP_1858-fw	GCA CTA GCT AGC CGC GGC CTC GAT TTT TAT AAA TCC	*Nhe*I	this study
P34	XbaI-PP_1858-GS-His6-rev	CGT CTA GAT TAG TGA TGG TGA TGG TGA TGC GAG CCC TTC TTG GAG CGG CCT TTG AA	*Xba*I	this study
P35	*efp Ppu* 29 30 31 OL fw	CGT AAC AGC GCG ATC ATG AAG ACC		this study
P36	*efp Ppu* K29A OL rev	CAT GAT CGC GCT GTT ACG GCC CGA CGC GGT GAA CTC AGC		this study
P37	*efp Ppu* K29R OL rev	CAT GAT CGC GCT GTT ACG GCC CGA GCG GGT GAA CTC AGC		this study
P38	*efp Ppu* S30A OL rev	CAT GAT CGC GCT GTT ACG GCC CGC CTT GGT GAA CTC AGC		this study
P39	*efp Ppu* S30G OL rev	CAT GAT CGC GCT GTT ACG GCC GCC CTT GGT GAA CTC AGC		this study
P40	*efp Ppu* S30P OL rev	CAT GAT CGC GCT GTT ACG GCC CGG CTT GGT GAA CTC AGC		this study
P41	*efp Ppu* G31A OL rev	CAT GAT CGC GCT GTT ACG CGC CGA CTT GGT GAA CTC		this study
P42	*efp Ppu* G31S OL rev	CAT GAT CGC GCT GTT ACG GCT CGA CTT GGT GAA CTC		this study
P43	*efp Ppu* R32K fw	ACC AAG TCG GGC AAG AAC AGC GCG ATC		this study
P44	*efp Ppu* R32K rev	GAT CGC GCT GTT CTT GCC CGA CTT GGT		this study
P45	*efp Ppu* 33 34 35 OL fw	ATC ATG AAG ACC AAG CTG AAG AAC CTG		this study
P46	*efp Ppu* N33D OL rev	CTT CAG CTT GGT CTT CAT GAT CGC GCT ATC ACG GCC CGA CTT		this study

P47	*efp Ppu* N33G OL rev	CTT CAG CTT GGT CTT CAT GAT CGC GCT GCC ACG GCC CGA CTT		this study
P48	*efp Ppu* S34A OL rev	CTT CAG CTT GGT CTT CAT GAT CGC CGC GTT ACG GCC CGA		this study
P49	*efp Ppu* S34Q OL rev	CTT CAG CTT GGT CTT CAT GAT CGC CTG GTT ACG GCC CGA		this study
P50	*efp Ppu* A35S OL rev	CTT CAG CTT GGT CTT CAT GAT GCT GCT GTT ACG GCC		this study
**Primers cross modification/activation and overproduction**				
P51	*efp Eco* domainI Ppu OL fw	AAG CTG GAC GAC GTG ATC CTG GAT ATG AAC CTG ACT TAC CTG		this study
P52	*efp Eco* domainI Ppu OL rev	CAG GTA AGT CAG GTT CAT ATC CAG GAT CAC GTC GTC CAG CTT		this study
P53	*efp Eco* loop *Ppu* OL fw	AAG TCG GGC CGT AAC AGC GCG TTT GCT CGC GTT AAA CTG CGT		this study
P54	*efp Eco* loop *Ppu* OL rev	CGC GCT GTT ACG GCC CGA CTT TAC GAA TTC ACT CGC TTC AAC		this study
P55	*efp Eco* P32S K34R OL fw	GAA TTC GTA AAA AGC GGT CGC GGC CAG GCA TTT		this study
P56	EF-P *Eco* P32S K34R OL rev	AAA TGC CTG GCC GCG ACC GCT TTT TAC GAA TTC		this study
P57	*efp Eco* K34R G35N OL fw	CCG GGT CGC AAC CAG GCA TTT GCT CG		this study
P58	*efp Eco* K34R G35N OL rev	CGA GCA AAT GCC TGG TTG CGA CCC GG		this study
P59	*efp Eco* K34R Q36S OL fw	CGC GGC AGC GCA TTT GCT CGC GTT A		this study
P60	*efp Eco* K34R Q36S OL rev	TAA CGC GAG CAA ATG CGC TGC CGC G		this study
P61	*efp Eco* P32S K34R G35N OL fw	AGC GGT CGC AAC CAG GCA TTT GCT CG		this study

P62	*efp Eco* P32S K34R G35N OL rev	CGA GCA AAT GCC TGG TTG CGA CCG CT		this study
P63	*efp Eco* P32S K34R Q36S OL fw	AGC GGT CGC GGC AGC GCA TTT GCT CG		this study
P64	*efp Eco* P32S K34R Q36S OL rev	CGA GCA AAT GCG CTG CCG CGA CCG CT		this study
P65	*efp Eco* K34R G35N Q36S OL fw	GGG TCG CAA CAG CGC ATT TG		this study
P66	*efp Eco* K34R G35N Q36S OL rev	CAA ATG CGC TGT TGC GAC CC		this study
P67	*earP So* rev	GCG GTA CCC GAT TTT CTA TTT CAG CGC AGC AT	*Kpn*I	this study
**Primers pET SUMO constructs**				
P68	*efp Eco*-SUMO-fw	ATG GCA ACG TAC TAT AGC AAC GAT TTT		this study
P69	*efp Eco*-SUMO-rev	TTA GTG ATG GTG ATG GTG ATG GCT		this study
P70	*efp Eco* K34R OL fw	GTA AAA CCG GGT CGC GGC CAG GCA TTT		this study
P71	*efp Eco* K34R OL rev	AAA TGC CTG GCC GCG ACC CGG TTT TAC		this study
**Primers isoelectric focusing and pBAD33-*epmA***				
P72	*efp-yjeA*-OL-XbaI-fw	CGT GAA GTA ATC TAG ATT GTC AAA AAC TGG AGA TTT AAC TAT GAG C	*Xba*I	this study
P73	*efp-yjeA*-OL-XbaI-rev	TTT TTG ACA ATC TAG ATT ACT TCA CGC GAG AGA CGT ATT CA	*Xba*I	this study
P74	*yjeA-yjeK*-OL-PstI-fw	GCA TAA CTG CAG GGT AGC TAA GCC ACA AAA TGG CG	*Pst*I	this study
P75	*yjeA-yjeK*-OL-PstI-rev	GCT ACC CTG CAG TTA TGC CCG GTC AAC GCT AAA G	*Pst*I	this study
P76	SacI-RBS-*efp*-fw	GCG ATG AGC TCA ATT AAC AAA TTT CAG AGG GCC TTA TGG	*Sac*I	this study

P77	SphI-*yjeK*-rev	GCA TCG CAT GCT TAC TGC TGG CGT AGC TGG AG	*Sph*I	this study
**Primer for strain construction of MG-CR-efp-epmA-KanR**				
P78	*epmA* fw	CAC CGC TGT TTG ATT CCT GCG T		this study
P79	*epmA* rev	GCT ACA GAA TGG CGC TTA T CA CG		this study
**Primer for for bacterial two-hybrid (BTH)**				
P80	XbaI-*efp*-*Eco* FW	GTA TCG TCT AGA GGC AAC GTA CTA TAG CAA CGA TTT TCG TG	*Xba*I	this study
P81	XmaI-*efp*-*Eco* Rev	GTA TCG CCC GGG ACT TCA CGC GAG AGA CGT ATT CAC C	*Xma*I	this study

References

Baba, T., Ara, T., Hasegawa, M., Takai, Y., Okumura, Y., Baba, M., et al. (2006). Construction of *Escherichia coli* K-12 in-frame, single-gene knockout mutants: the Keio collection. *Mol. Syst. Biol.* 2**,** 2006 0008. doi:10.1038/msb4100050

Datsenko, K.A., and Wanner, B.L. (2000). One-step inactivation of chromosomal genes in *Escherichia coli* K-12 using PCR products. *Proc. Natl. Acad. Sci. USA* 97**,** 6640-6645. doi:10.1073/pnas.120163297

Fried, L., Lassak, J., and Jung, K. (2012). A comprehensive toolbox for the rapid construction of *lacZ* fusion reporters. *J. Microbiol. Methods* 91**,** 537-543. doi:10.1016/j.mimet.2012.09.023

Guzman, L.M., Belin, D., Carson, M.J., and Beckwith, J. (1995). Tight regulation, modulation, and high-level expression by vectors containing the arabinose P_{BAD} promoter. *J. Bacteriol.* 177**,** 4121-4130.

Karimova, G., Dautin, N., and Ladant, D. (2005). Interaction network among *Escherichia coli* membrane proteins involved in cell division as revealed by bacterial two-hybrid analysis. *J. Bacteriol.* 187**,** 2233-2243. doi:10.1128/JB.187.7.2233-2243.2005

Kovach, M.E., Elzer, P.H., Hill, D.S., Robertson, G.T., Farris, M.A., Roop, R.M., 2nd, et al. (1995). Four new derivatives of the broad-host-range cloning vector pBBR1MCS, carrying different antibiotic-resistance cassettes. *Gene* 166**,** 175-176.

Krafczyk, R., Macosek, J., Jagtap, P.K.A., Gast, D., Wunder, S., Mitra, P., et al. (2017). Structural Basis for EarP-Mediated Arginine Glycosylation of Translation Elongation Factor EF-P. *mBio* 8**,** e01412-01417. doi:10.1128/mBio.01412-17

Lassak, J., Henche, A.L., Binnenkade, L., and Thormann, K.M. (2010). ArcS, the cognate sensor kinase in an atypical Arc system of *Shewanella oneidensis* MR-1. *Appl. Environ. Microbiol.* 76**,** 3263-3274. doi:10.1128/AEM.00512-10

Lassak, J., Keilhauer, E.C., Fürst, M., Wuichet, K., Gödeke, J., Starosta, A.L., et al. (2015). Arginine-rhamnosylation as new strategy to activate translation elongation factor P. *Nat. Chem. Biol.* 11**,** 266-270. doi:10.1038/nchembio.1751

Macinga, D.R., Parojcic, M.M., and Rather, P.N. (1995). Identification and analysis of *aarP*, a transcriptional activator of the 2'-N-acetyltransferase in *Providencia stuartii*. *J. Bacteriol.* 177**,** 3407-3413.

Peil, L., Starosta, A.L., Lassak, J., Atkinson, G.C., Virumae, K., Spitzer, M., et al. (2013). Distinct XPPX sequence motifs induce ribosome stalling, which is rescued by the translation elongation factor EF-P. *Proc. Natl. Acad. Sci. USA* 110**,** 15265-15270. doi:10.1073/pnas.1310642110

Studier, F.W., and Moffatt, B.A. (1986). Use of bacteriophage T7 RNA polymerase to direct selective high-level expression of cloned genes. *J. Mol. Biol.* 189**,** 113-130.

Ude, S.C.M. (2013). *The role of elongation factor EF-P in translation and in copy number control of the transcriptional regulator CadC in Escherichia coli.* Dissertation, LMU München: Faculty of Biology.

Volkwein, W., Maier, C., Krafczyk, R., Jung, K., and Lassak, J. (2017). A versatile toolbox for the control of protein levels using N_{ε}-acetyl-L-lysine dependent amber suppression. *ACS Synth. Biol.* 6**,** 1892-1902. doi:10.1021/acssynbio.7b00048
